# Plant-Mediated Production of Antibacterial Nanomaterials Fulfilling a Reactive Oxygen Species Strategy

**DOI:** 10.3390/ijms27177895

**Published:** 2026-09-04

**Authors:** Olga Tsivileva

**Affiliations:** Institute of Biochemistry and Physiology of Plants and Microorganisms, Saratov Scientific Centre of the Russian Academy of Sciences, Saratov 410049, Russia; tsivileva_o@ibppm.ru

**Keywords:** nanomaterials, metal nanoparticles, phytochemical, herbal extract, phytosynthesis, pathogenic bacteria, antimicrobial activity, antibacterial action mechanism, ROS

## Abstract

Innovative antibacterial agents, which need to be urgently developed, should provide a new path that avoids pathogenic bacteria drug resistance. A promising strategy to treat bacterial infections consists of implementing nanoparticles (NPs). Several nanomaterials, including metal NPs, induce oxidative stress in living cells by producing reactive radicals at their surface. The oxidative stress thus raised is a potent mechanism of antibacterial action. Owing to their reactive oxygen species (ROS)-related ability, metal NPs could contribute to the targeted bioactivity of preparations designed on their basis. However, totally inorganic nanomaterials frequently suffer from relatively low selectivity with respect to bacterial targets, limited dispersibility, and undesirable side effects linked to ambiguous toxicity. For the development of bacteria drug resistance, the incorporation of specific ligands responsible for bacterial eradication into NPs seems to be necessary. The synergistic action of NPs with natural materials demonstrates complementary effects and improved antibacterial capacity. The modification of NPs by natural organic supporting agents extracted from plants could potentially resolve some of the existing issues related to bacterial drug resistance. The purpose of the current work is to review the ROS-mediated antibacterial manifestation strategy of metal NPs conjugated with botanical bactericidal substances. Different aspects of the metal toxicity of NPs and the compromised selectivity of ROS for bacterial pathogens are also considered.

## 1. Introduction

Microbial spoilage and oxidative damage are among the major problems that continuously poison and impede the development of crop production, followed by concomitant challenges for humans. Generally, natural plant products have played an essential role in drug design and managing human diseases over thousands of years [1]. The cost efficiency and local availability of natural plant material have made it a sustainable source of drugs. The pharmaceutical agents derived from plants play a pivotal role in realizing therapeutic approaches owing to their chemical diversity and various biological activities against a lot of diseases’ causative agents. Many of the latter are bacterial in nature. Over the last few decades, plant metabolites have gained more importance in mediating the synthesis and manufacture of antibacterial compounds [2,3].

Oxidative stress is currently accepted as a potent mechanism of antibacterial action. The production and elimination of reactive oxygen species (ROS) are normally controlled by fine cellular mechanisms, and a stable level of ROS is present in cells. The equilibrium between ROS generation and degradation is disturbed as a result of their increased concentrations, referred to as oxidative stress [4]. In bacterial cells, oxidative stress occurs when accumulated ROS exceed a threshold of cellular tolerance to them. An increase in the level of ROS efficiently assists in destroying pathogenic cells or even in eradicating persisting cells in bacterial biofilms [5]. Engineered nanoparticles (NPs), including metal and metal oxide NPs, induce oxidative stress in cells by producing reactive radicals at or near their surface [6]. By enhancing the bacterial cell wall/membrane damage rate, intracellularly accumulated NPs in pathogens ultimately lead to cell death. Green synthesis of NPs is an economic method and has immense potential for their industrial manufacturing [7]. Such biofabrication is eco-friendly and much cleaner, simpler, and less toxic than chemical process-oriented manufacturing [8]. Parts of many nutritional and medicinal plants are appropriate for use in preparing extracts containing metabolites with metal-cation-reducing and NP-stabilizing abilities. Plant-mediated NP preparation techniques provide benefits over physicochemical synthetic processes as they are readily accessible, ecosafe, and mostly inexpensive [9]. This review aims to consider metal NPs from the viewpoints of their biomediated (by plant metabolites) production, the ROS-mediated strategy they implement to eradicate pathogenic bacteria, and future prospects of their application.

## 2. Metal NPs Potent for Killing Bacteria Through ROS-Mediated Pathways

The application of metal NPs attracted attention due to their significance as delivery systems [10] within the framework of indirect antibacterial effects. Metal-based nanostructures have also emerged in the field of directly applicable tools used to control pathogens. Regarding their superior Fenton activity [11], these were typically represented by ferromagnetic NPs, complemented by other metal-sourced NPs, iron-doped metal oxide NPs [12], totally inorganic nanohybrids with matrix-integrated [13] or encapsulated [14] NPs, photocatalytic nanosized formulations containing titanium dioxide doped or not with zinc [15], and other kinds of complex-structured nanomaterials characterized by antimicrobial properties. The ability to induce ROS formation is one of the bactericidal mechanisms of NPs [4]. The majority of metal NPs primarily affect cell wall and membrane components [16]; selected NPs occur in the cytoplasm and cause cell instability, disruption, and oxidative stress. Their small particle size makes it possible for Ti-containing NPs to pass through the bacterial surface to perform lysis. The size of an NP is expected to modulate nanotoxicity, since ultrafine metal NPs were very frequently found to be much more hazardous than larger-sized NPs. Hence, the physicochemical variables of a given nanomaterial are subject to regulation in order to balance its possible toxicity and diminish unintended impacts on the ecosystem and its targeted bactericidal properties (Table 1).

It is important to note that the values presented vary significantly across studies. These disparities are caused by differences in test microbial systems, synthesis conditions (such as pH, temperature, and precursor concentration), NP size and morphology, and experimental procedures used to evaluate performance. As a result, these numbers should not be viewed as globally comparable measurements but rather as system-specific markers of antibacterial activity. The diverse data highlight the existing absence of standardized benchmarking techniques in biogenic metal NPs, including missing NP sizes, MIC values, and active compound specifications, which prevents direct comparisons between investigations. This lack of protocol standardization with respect to biogenic metal NPs undermines the ability to conduct systematic performance comparisons [38].

Metal-containing nanomaterials for the prevention and treatment of infections have advanced rapidly [39]. Novel antibacterials must be urgently developed to provide a new path for avoiding bacterial resistance. This path may rely on Fenton catalytic chemistry, i.e., reactions that specifically decompose hydrogen peroxide H_2_O_2_ to produce highly toxic hydroxyl radical anions, the most harmful ROS, to kill bacteria by oxidative stress [40,41]. However, the traditional Fenton reaction that implements the Fe^2+^/Fe^3+^ redox cycle, called iron Fenton-like systems [42], suffers from several drawbacks that limit its antibacterial efficiency. Nanotechnology has progressed rapidly toward iron-free Fenton-like systems to develop innovative nanomaterials with beneficial Fenton reactivity, also known as peroxidase-mimic nanoenzymes. A Fenton-like pathway for ROS production was well studied in metal NPs [43].

Potential antibacterial properties of some nanomaterials, such as metal (Ag, Au) and metal oxide nanoparticles (silver(I) oxide Ag_2_O, titanium dioxide TiO_2_, copper(II) oxide CuO, zinc oxide ZnO, magnesium oxide MgO), have been reported to be efficient against both Gram-positive and Gram-negative bacteria [4,44,45,46,47]. Since these NPs are subject to engineering design, including size adjustment, surface modification, and transformation into stimulus-responsive preparations, functionalized AgNPs, Au clusters, and ZnO and CuO nanomaterials demonstrate relatively high antibacterial effectiveness comparable to antibiotics and specialized drugs [16].

However, ROS delivery methods mediated by NPs lack desirable selectivity for pathogens, causing side effects in host tissues [4]. Despite the profound antibacterial activity of several nanomaterials capable of converting H_2_O_2_ to detrimental hydroxyl radicals even in neutral environments, many studies suggest that certain NPs can potentially trigger an enhancement in bacterial resistance. Furthermore, floating metal NPs tend to aggregate together and increase in particle size, especially in high-salt solutions. This drawback may reduce the NP–bacteria interface and hence the antibacterial potential. By applying organic compounds as the NP-stabilizing support, the antibacterial effect of metal NPs could be recovered, owing to a dispersing effect [13]. Provided that these organic compounds are botanical bactericidal agents, synergistic effects between inorganic (NPs) and organic components may occur with respect to the enhanced antibacterial trait. Importantly, the acting concentration of eco-harmful metal NPs can be drastically reduced by dilution with organic phases during the preparation of these biocomposites. Thus, the acting level of metal NPs in hybrids could be at a rate of a few mass percent of the overall composition weight.

Metal/metal oxide NPs can act as a trigger of oxidative stress [48]. NPs release metal cations and impair cells, thus enhancing ROS formation. Regarding the interaction between NPs and biomolecules, metal complexation occurs readily to form highly reactive complexes [48,49] responsible for increasing free radical species that mediate the enhanced generation of ROS. Highly reactive species, such as superoxide radical anions and hydroxyl radicals, further maintain oxidative stress onset in pathogens either directly or through the activation of enzymatic processes [50].

Moreover, metal NPs bound to proteins mimic signaling molecules, thus triggering diverse cellular processes, including ROS production [50] followed by induced cell toxicity, membrane injury, and disruption of vital cellular macromolecules [51]. A few metal oxide NPs, such as oxides and mixed oxides of cerium, manganese, cobalt, and iron, in which the chemical element is available in varied valence states, have the ability to function as nanozymes. Rapid redox exchange between different oxidation states of the given metal is critical for the intrinsic enzyme-like activity of NPs. The issue of environmentally oriented applications of nanozymes, including the destruction of multidrug-resistant bacteria, has become a hot point in academic study nowadays. ROS-generating nanozymes, which are nanomaterials with enzyme-like activity, can circumvent bacterial resistance mechanisms because, unlike antibiotics, they cannot target specific metabolic pathways [51]. Despite their advantages, nanozymes are generally toxic to both bacteria and mammalian cells [50]. The primary barrier to establishing biogenic nanocatalysts as a standardized technology is the intrinsic variability in plant systems and metabolites, which complicates the reproducibility of physicochemical characteristics. The notable absence of quantitative comparisons between the performance of biogenic catalysts and conventional catalysts using standardized metrics is further complicated by a lack of long-term stability studies under actual operational conditions [52]. These factors collectively hinder our ability to conclusively determine whether biogenic catalysts outperform synthetic analogues under equivalent conditions. Ultimately, direct one-to-one quantitative comparisons are impeded. In general, the evolution of biogenic NPs from laboratory curiosities to industrial solutions is hindered by underdeveloped catalytic benchmarking.

When comparing chemical nanozymes with biogenic nanocatalysts, one should distinguish between the metal core’s physical dimensions and its effective hydrodynamic behavior in the reaction medium. The hybrid nature of plant-engineered catalysts, particularly the organic–inorganic interface, is highly responsive to external stimuli [53]. Biogenic catalysts maintain this equilibrium, allowing for continuous catalytic turnover under favorable reaction conditions. This mechanistic elegance mimics enzymatic precision, allowing complex green transformations to occur under milder conditions than those of traditional industrial catalysts.

To better understand the mechanistic behavior of phytochemical-engineered nanocatalysts, several quantitative parameters are commonly used to describe catalytic pathways and efficiency. These parameters include electron transfer kinetics, active site density, and adsorption characteristics, which collectively determine catalytic performance and selectivity. Selectivity values are highly reaction-dependent and are frequently reported as representative ranges rather than universal constants. The published values of electron transfer rates in biogenic and nanostructured catalytic systems are typically derived from time-resolved spectroscopic or electrochemical techniques and vary significantly depending on catalyst composition, reaction environment, and measurement methodology. More and more authors note that the term “green” is often used without considering net ecological impact [54]. The field requires more specific evaluation standards and improved database construction to advance from passive evaluation to active sustainable design strategies.

Therefore, metal-containing NPs have a number of useful characteristics that can be applied in research and practice, including biotechnology. In particular, their high redox activity (as biocatalysts of redox reactions), ability to exert biocidal action against pathogenic bacteria, ability to penetrate the cell wall, thereby influencing target bacteria not only by means of membrane deformation, and other traits [55,56] are increasingly used. It is imperative to understand how biologically produced NPs exert regulatory effects on bacteria. The function of phytosynthesized metal NPs depends not only on the type, size, and acting concentration but also on plant components and experimental conditions.

## 3. Prerequisites for Using Plant Secondary Metabolites in the Phytosynthesis of Antibacterial Metal NPs

### 3.1. Phytosynthesis Evidenced by Defensive Function of Phytochemicals

To withstand pathogenic invasion, plants act via biosynthesis of secondary metabolites [57]. Constitutive chemical compounds stored in plants in inactive form or their pre-existing precursors (phytoanticipins), along with compounds induced by a plant response to pathogen challenge (phytoalexins), are referred to as secondary metabolites with defense purposes, among other functions [5]. Trends in utilizing plant metabolites to control bacterial pathogens include both direct antibacterial action and indirect plant resilience [58], since most compounds synthesized in response to pathogen attacks might have regulatory functions, indirectly increasing the level of plant resistance. Employing isolated phytochemicals and crude herbal extracts that act directly on target harmful species lays a basis for a bacterial management strategy.

There is a tendency to distinguish three extensive chemical classes of plant secondary metabolites as natural products with defensive functions: terpenes and terpenoids (about 55%), alkaloids (about 27%), and polyphenolic compounds (about 18%) [58]. Polyphenolic compounds are divided in turn into four major groups: phenolic acids, flavonoids, stilbenes and lignans [59]. Relative chemical structures are most frequently used by plants in related families, although some compounds serve as chemical defense across taxa. These secondary metabolites provide plants with bioactivities that underlie their diverse therapeutic potentialities. The multicomponent nature of antibacterial plant formulations capable of affecting multiple targets is summarized in Table 2.

**Figure 1:**
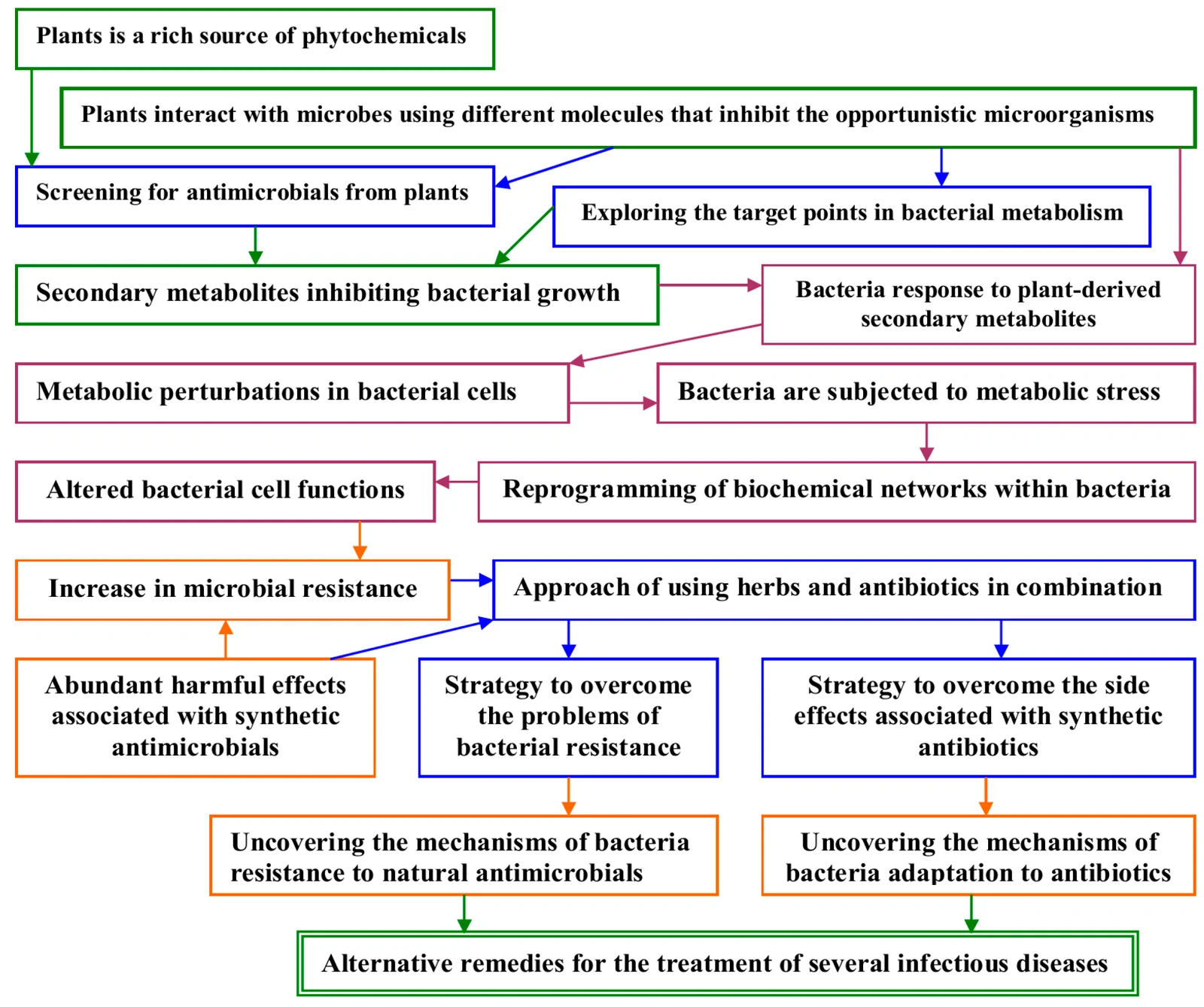
Schematic outlook of the challenges involved in studying plants as a source of antimicrobial compounds that impact bacterial biochemical processes.

Numerous published works discuss plant antibacterial potential; however, only a limited number of works elucidate the mechanisms of plant-derived agents that function against bacterial species [77]. The antibacterial potential of plant extracts is impacted by several factors and realized through different pathways. The potential antimicrobial activity of plant-derived substances relies to a large extent on synergistic interactions between the constituents of the source herbal product. Synergy is vitally important to explain the low-dose effect on bacteria [78]. Interactions between different bioactive components in plant extracts and distinct molecular targets in pathogens promote synergistic effects [79]. Combined use of plant extracts might improve pharmacological action by attaining multiple targets simultaneously, reducing the specific amounts of individual components, and thereby lowering side effects [80]. The components of the principal approach to discovering efficient antimicrobials in plants [3] are depicted in Figure 1.

Renewed interest in natural products has been raised regarding plant essential oils, valued for their ability to prevent the emergence of “antibiotic resistance”, a phenomenon caused by the long-term use of synthetic antibiotics [81]. Recent studies have confirmed the antibacterial efficiency of essential oils against a range of microbes [74]. Synergistic bactericidal effects of the most potent medicinal plant essential oils in combination with some antibiotics have been reported. Data on the antibiotic-enhancing activity of many plant essential oils testify to a rather high potential use of these alternative remedies against clinical multi-resistant bacteria [81]. Previous results on the synergism between essential oils and common synthetic antibiotics have been affirmed by more recent research [74,82]. Through incorporation of tea-tree or lavender oils with a broad-spectrum fluoroquinolone ofloxacin in a hydrogel matrix, without any appreciable interaction with the latter, severe adverse effects on common bacterial pathogens in wound infections were revealed. This technique, based on combined encapsulation of an essential oil and an antibiotic, reported for the first time by Mahmood et al. [82], has been further developed. Eucalyptus oil and oleic acid were incorporated into specially designed sodium alginate-based microbeads [83]; an absence of interactions between the macrolide antibiotic clarithromycin and the bead polymers was confirmed, and synergistic antibacterial effects against both Gram-positive and Gram-negative bacterial strains were demonstrated. Alpha-linoleic acid and palmitic acid were key active compounds identified while studying the antimicrobial potential of *Solanum xanthocarpum* fruit extracted with methanol and subsequently applied against methicillin-resistant *Staphylococcus aureus* [75].

Alternative therapeutic strategies based on promising antimicrobial plant biomass are increasingly recognized [84] and provide an opportunity to combat resistant pathogens owing to the presence of bioactive phytoconstituents [75]. Favorable features with appreciable antibacterial activity have been demonstrated mainly by polyphenols, encompassing flavonoids, as well as by terpenes and their oxygenated derivatives.

**Figure 2:**
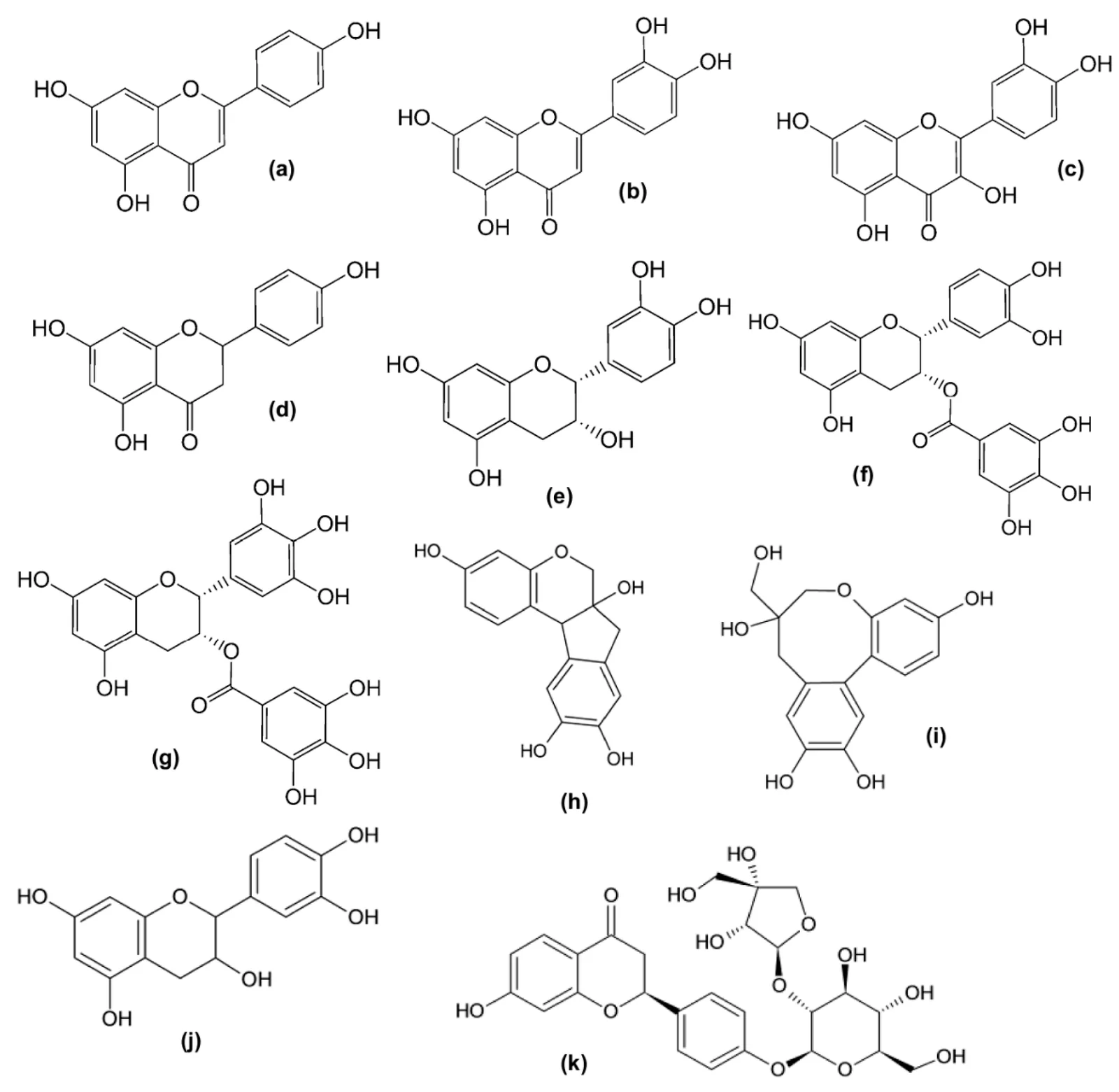
Examples of the chemical structures of flavonoids studied extensively for their antimicrobial properties: (**a**) apigenin; (**b**) luteolin; (**c**) quercetin; (**d**) naringenin; (**e**) epicatechin; (**f**) epicatechin-3-gallate; (**g**) epigallocatechin-3-gallate; (**h**) brazilin; (**i**) protosappanin B; (**j**) catechin; (**k**) liquiritin apioside.

Flavonoids are important defense molecules, representing a class of aromatic secondary metabolites considered as one of the adaptive plant responses to adverse environments, including bacterial infections. Flavonoid biosynthesis and variety are impacted by appropriate stress conditions [85]. Flavonoids have attracted serious consideration from the pharmaceutical industry [86], as they are also potent antimicrobial agents against a wide range of pathogenic microorganisms. These bioactive polyphenols are essential secondary plant metabolites that are abundant in the human diet. The flavonoid structure incorporates a phenyl-benzopyrane moiety (carbon core C6-C3-C6 with a basic structure of 2-phenyl-benzo-γ-pyrane) [86]. In this moiety, a benzene ring A is fused with a heterocyclic pyran or pyrone ring C, which in turn is bound to the second benzene ring B. The basic flavonoid structure can undergo different substitution patterns, leading to countless chemical derivatives exemplified by the compounds in Figure 2.

It was proposed that flavonoids play an important role in the response to pathogen attack and could essentially be considered phytoalexins [87]. Flavonoids exert multiple effects on plant defenses, but they are also involved in the therapy of many human pathologies via different mechanisms, such as oxidative stress modulation or direct interaction with cell wall and membrane components [88]. To combat phytopathogens, plants mobilize distinct layers of defense responses implemented in innate immunity, including flavonoid biosynthesis [86], resulting in impaired bacterial homeostasis, motility, and biofilm and toxin production [89].

Flavone apigenin (Figure 2) and its glycosylated derivatives exuded from rice (*Oryza sativa* L.) plant roots induced root colonization by soil diazotrophic bacteria and the subsequent formation of biofilms. These beneficial bacterial biofilms create microaerophilic conditions that favor the activity of bacterial nitrogenase, resulting in improved nutrient conditions for rice plants [90]. Evidence of stimulated biological nitrogen fixation after modifying the flavone apigenin biosynthetic pathway in hexaploid wheat (*Triticum aestivum*) plants was also provided [91].

Flavonoids brazilin, protosappanin B, liquiritin apioside, and catechin were reported to have various antibacterial mechanisms against pathogenic bacteria [80]. These substances (Figure 2) were capable of inducing injury in the bacterial respiratory chain, membrane proteins, and lipid bilayer. The antibacterial properties of flavonol quercetin, flavanone naringenin, and flavanol catechin (Figure 2) were also rather profound. Flavonoids inhibited *Staphylococcus aureus*-induced hemolysis and modified the membranes of bacterial cells and erythrocytes [88].

Along with flavonoids, a chemical class of terpenes represents one of the major groups of phytoconstituents that has been mostly studied for antibacterial properties. Terpenes and their oxygen-containing derivatives are recognized for their antibacterial traits that are efficient against a variety of pathogens [84]. Monoterpene 1,8-cineole (1,3,3-trimethyl-2-oxabicyclo[2.2.2]octane), also known as eucalyptol, derived from botanical sources such as eucalyptus (*Eucalyptus globulus*), rosemary (*Rosmarinus officinalis* L.), and camphor laurel (*Cinnamomum camphora* (L.) Ness & Eberm.), exhibits an array of biological activities, including antibacterial ones useful for treating a variety of respiratory and digestive diseases, as well as neurological disorders [63]. A substantially diminished minimum inhibitory concentration (MIC) of antibiotics applied jointly with eucalyptol was observed [92]. Eucalyptol derived from eucalyptus oil displayed broad-range antagonistic activity, specifically against the respiratory pathogens *Streptococcus pneumoniae* and *Haemophilus influenzae*, resulting in inhibited bacterial biofilm growth and reduced virulence. A very high level of eucalyptol, a versatile phytoconstituent with pharmaceutical applications [93], was reported for *Eucalyptus nicholii*, whose essential oil contained up to 90% of 1,8-cineole. The antibacterial activity of this monoterpene was proposed to be related to its ability to induce cell membrane permeabilization or to its hydrophobic nature, affecting pathogens comprising an outer lipopolysaccharide membrane [66].

Oxygenated sesquiterpenes (Z)-α-santalol and (Z)-β-santalol, the dominant constituents of *Santalum album* essential oil, can be applied in food preservation owing to their considerable antibacterial potentialities [74]. *Mentha viridis* (mint) from the family Lamiaceae is known worldwide for its medicinal and aromatic properties. Mint essential oils isolated from leaves have high commercial value owing to monoterpene menthol, which shows antibacterial activity [70]. *Mentha piperita* L. (peppermint) is a famous pharmaceutical herb. Antimicrobial activity is among the main pharmacological properties of this phytoresource, including its extracts and isolated essential oils. The principal component of the latter is usually (−)-menthol, with smaller amounts of the menthol stereoisomers (+)-*neo*-menthol and (+)-*iso*-menthol [69].

To draw a connection with the inorganic component (metal NPs) of the antibacterial nanocompositions in question, it should be noted that when tested separately, plant-derived antimicrobials might be deemed inefficient [88,94], which underscores the need for further investigation of a synergistic interaction between plant metabolites and metal NPs as proven antibacterial agents.

### 3.2. Phytosynthesis Evidenced by Structural Attributes of Phytochemicals

The therapeutic activity of drugs impaired by the bacterial resistance mechanism could potentially be restored by plant products that act as antibiotic adjuvants [5], which are considered to be perfect antibacterial therapeutic candidates. Botanical antimicrobials cause cell morphology distortion, membrane structure destabilization, and increased membrane fluidity and permeability, which allows one to consider the cell membrane as the primary target of antibacterial impact by many phytochemicals. This impact is enhanced when antimicrobials are conjugated with metal-based nanostructures [95].

There has been a constant increase in research interest in the use of metal-containing NPs obtained by so-called green nanosynthesis, i.e., using either biological objects for their manufacturing or natural extracts, juices and homogenates [96,97,98,99]. Plant-mediated synthesis of NPs is the most widely used and easiest biosynthesis approach [100]. Phyto-NPs are relatively biocompatible for biological applications owing to the minimized level of hazardous chemicals involved in their production. In particular, the design of nanopharmaceuticals comprising a flavonoid moiety conjugated with a nanosized delivery platform may resolve the problems of intrinsically reduced chemical stability, low solubility, and poor absorption of flavonoids; it may also enhance the specificity of their effects [101].

The synthesis of metal NPs should consider the diversity of plants and their metabolites from the viewpoint of metal ion-reducing and stabilizing capacity to improve concentration, provide a broader range of phytochemicals in crude solvent extracts, and efficiently reduce metal salts into NPs [102]. Dried plant biomass is used as a bioreducing agent and a precursor in the general process that uses plant metabolites to make metallic NPs [103]. Safe raw materials for bioproducing NPs include nutritional plant fruits, which are free of toxicants and enriched with reducing bioproducts [34]. Thus, green approaches avoid costly chemicals and toxic byproducts, utilize less energy, and result in more eco-friendly antibacterials. Green synthesis of metal NPs is generally far superior to chemical synthesis.

A lot of studies reported the application of different plant extracts for the phytosynthesis of metal NPs. Extracts from different parts of plants, such as stems, roots, fruits, seeds, peels, leaves, bulbs, gum, husks, pods, rhizomes, cloves, bark and flowers, were considered to be appropriate as potent reducing and stabilizing agents for metal NPs [104]. Naturally, NPs producers should take into account the variability in such plant materials, including plant harvesting season and varied phytochemical composition, as these factors influence the synthesis and activity of NPs. Synthesis mechanisms differ significantly even within the same plant species. Parameters such as pH, temperature, and precursor concentration dramatically alter the resulting NP properties. Therefore, the green synthesis literature exhibits major deficiencies in several areas essential for rigorous evaluation of bioproduced nanomaterials. In many studies, the reported performance is based on the total mass of an organo-metallic composite rather than the actual active metal content. The presence of a bio-capping layer introduces additional uncertainties.

Plant metabolite-engineered metal NPs feature a complex hybrid architecture with an inorganic crystalline core enclosed within an organic, amorphous bioshell. Phyto-originated substances provide a robust structural framework, often composed of polysaccharides, which keep individual NPs in a fixed spatial orientation [105,106]. Polysaccharidic biological scaffolds impose rigid physical templates on the formation of metal NPs, thereby limiting particle size distribution and reducing their polydispersity. This structural confinement [53] regulates NP nucleation and growth, suppresses agglomeration, and provides intrinsic stabilization comparable to synthetic capping agents. The role of confinement in dictating electronic interactions between metal cores and biological functional groups represents a critical gap in our current understanding of NP biosynthesis. Plant-derived polysaccharide-rich matrices facilitate biosorption and reduction, with reducing sugars acting as electron donors for metal ion conversion [107]. AgNPs prepared using starch as a reducing agent were spherical with a diameter below 10 nm [108]. Green biosynthesis of flower-shaped AgNPs was attained using starch (corn, cassava, and sago) as the source of the reducing-sugar agent, where glucose reduces Ag(I) from silver nitrate into the zero oxidation state in AgNPs [38]. Biosynthesis is influenced by a myriad of factors, such as pH, temperature, reactant concentrations, reaction time, light, etc. The physicochemical factors associated with the synthesis process affect the morphological, catalytic, and biological properties, including antibacterial, of the NPs produced. Variations in surface properties dependent on plant metabolites implemented in biofabrication were found to influence the performance of metal NPs in antimicrobial applications [107].

Metal cations can be bioreduced to NPs by utilizing the hydroxyl and carbonyl functionalities of plant secondary metabolites. Efficient reduction of precursor metal-containing substances to NPs is mediated by phenols and other phytochemicals, whose molecular frameworks form a stabilizing organic matrix that wraps around NPs to induce reliable stability against agglomeration. These processes are favored by the fact that plants contain high amounts of antioxidant polyphenols, including flavonoids [109,110]. The ability of plant polyphenols to complex with polymers and metals appears to be the basis of their inhibitory effect on bacteria. In the course of the direct interaction between polyphenols and microorganisms, the mechanisms underlying the antibacterial activity of flavonoids include metal ion chelation and ROS generation [86,111]. Flavonoids actively reduce chelated metal ions into nanoparticles [112,113]. For instance, quercetin and apigenin (Figure 2) form chelates with many transition metal ions to yield complexes with renewed biological properties applicable to pathogenic-bacteria-fighting techniques [111].

Spatially adjacent hydroxyl groups attached to the molecular backbone have been demonstrated to be of special importance in the antibacterial activity of a given compound [114]. Catechins, with an ortho-diphenol (catechol) functional moiety, have been broadly examined for their antibacterial traits against both Gram-positive and Gram-negative bacteria. Furthermore, several works have highlighted the pro-oxidative ability of polyphenols previously described as antioxidants, for instance, the flavanols epicatechin, epigallocatechin gallate, and flavonol quercetin [86] (Figure 2). Reportedly, the bacteria-fighting action of catechins relies on an oxidative burst initiated by ROS production and the subsequent membrane injury [115]. However, high concentrations of catechins were required for the manifestation of antibacterial effects, which were less effective against Gram-negative bacteria than against Gram-positive bacteria. In this regard, there exists a need to invent pharmaceuticals capable of withstanding both Gram-positive and Gram-negative bacterial pathogens. Such pharmaceuticals could be designed based on flavonoids modified with bioactive molecules [111] or with metal NPs. Compromised bacterial ability to cope with oxidative attack from common antibiotics was proposed to be further decreased by endogenous ROS generation by flavonoids, impairing the bacterial antioxidative defense system and eventually enhancing antibiotic efficiency [86].

## 4. Metal–Phytochemical Antibacterial Nanocompositions

### 4.1. Silver–Phytochemical Antibacterial Nanocompositions

Silver nanoparticles (AgNPs) obtained by chemical or green nanosynthesis have been increasingly used in research and practice in recent years owing to their acceptable biocompatibility and moderate toxicity at a relatively low concentration [116]. One of the key questions is how AgNPs obtained by eco-friendly methods modify bacterial growth in various environments and biotechnologically oriented systems.

AgNPs obtained using plant extracts demonstrated high antimicrobial activity against Gram-positive bacteria (*Staphylococcus aureus*, *Bacillus subtilis*) and Gram-negative bacteria (*Escherichia coli*, *Pseudomonas aeruginosa*) [117]. For instance, Kumari et al. [118] studied the interrelated effects of coated AgNPs against *Shigella sonnei*, *Pseudomonas aeruginosa* and *Staphylococcus aureus*. The effects included an increase in oxidative stress followed by severe damage to the bacterial membrane, subsequent reinforced AgNPs uptake, further injury to bacterial cells, and death of pathogens [118]. This research also showed that the biologically produced AgNPs surface-modified with antimicrobial metabolites were more frequently internalized by bacteria and more effective at killing pathogens compared to chemically synthesized NPs.

It has been shown that AgNPs can penetrate the cell wall and outer membrane of bacteria to enter the cytoplasm, and they can also form large agglomerates inside the cell wall near the cell membrane that release silver cations, gradually exerting a prolonged bactericidal effect [119]. Inside a bacterial cell, by binding with genetic material, AgNPs can inhibit transcription and translation processes [96]. In addition, silver forms chemical bonds with sulfhydryl groups of bacterial proteins, causing denaturation of the latter [120]. Smaller-sized AgNPs, owing to their larger ratio of surface area to overall volume, exhibit higher bactericidal activity [121]. Along with the mentioned antibacterial mechanisms, nanosized silver causes the formation of ROS and initiates mechanical damage to membrane lipids and their peroxidation, additionally promoting the penetration of NPs into the cell [122]. This can result in physiological and biochemical changes in the cellular environment, damaged respiratory chains, disturbed cell division, and so on. Free radical generation under the AgNP–bacterium interaction finally leads to cell death [123].

The successful synthesis of AgNPs was realized using a pomegranate peel extract [124]. Pomegranate peel is an excellent source of polyphenols, such as tannins, ellagic acid, gallic acids, etc. It was stated that the composition of these substances acts as both reducing and capping agents to yield AgNPs from a silver-containing precursor, and the Ag complex may be a result of a coordination process involving Ag cations and the acidic groups of polyphenols [125]. Compared to commonly discussed reductive and capping plant-sourced phenolics, there are few reports on the synthesis of AgNPs mediated by bioreduction induced by purified flavonoids. For thousands of years, *Ocimum sanctum* (Tulsi) phytonutrients have exhibited therapeutic benefits owing to their medicinal properties. The leaf extracts of *Ocimum sanctum* contain the flavonoid quercetin as the major biomolecule [126]. Quercetin reacts with silver cations via the most reactive hydroxyl groups attached to carbon atoms of the aromatic ring [127], reduces Ag^+^ to AgNPs, and provides stability against agglomeration. Furthermore, the enzymes present in plant materials interact with silver cations to form an enzyme–substrate complex with charge transfer between quercetin and these cations, resulting in AgNPs capped with protein. Ultimately, quercetin-stabilized, biomacromolecule-capped AgNPs possess enhanced antibacterial activity against Gram-negative bacterial strains of *E. coli* compared to AgNPs synthesized separately through pure *Ocimum sanctum* leaf extract or purified quercetin [126].

Purified secondary metabolites, including the flavonoids hesperidin, naringin and diosmin, have been used separately to synthesize AgNPs [94]. Hesperidin and naringin are flavanone glycosides; both have a saturated pyran ring in their chemical structures. Whereas diosmin, a flavone glycoside derived from the citrus-sourced flavanone hesperidin, has an unsaturated pyran ring (with a double bond), which may explain the dissimilar behavior of diosmin compared with hesperidin and naringin. These AgNPs display relatively low bactericidal effects against common pathogens, including *Escherichia coli*, *Pseudomonas putida* and *Staphylococcus aureus*. Purified flavonoids serve as capping agents for AgNPs, which could explain the decreased antibacterial activity affected by hesperidin, naringin and diosmin. Biogenic NPs, unlike their chemically produced counterparts, are naturally stabilized by a complex organic capping layer. This layer does more than just stabilize and reduce metal toward its zero oxidation state; it also creates a distinct chemical microenvironment that alters biological properties, including antibacterial activity. The biogenic interface is distinguished by a capping layer that acts as an advanced molecular filter [53]. This biological shell is thought to influence the reactive molecule accessibility and protect active sites from deactivation, allowing small reactant species to diffuse through the organic matrix to metallic active sites while also preventing larger, bulky macromolecules from damaging or blocking the metal’s active components [53,104]. Therefore, this natural architecture, functioning via selectivity and protection, could attenuate some interactions between NPs and bacterial (macro)molecules, thus decreasing antibacterial activity. It should be noted that the field faces significant hurdles in batch-to-batch reproducibility and standardized reporting of metal-normalized activity. In particular, achieving reproducibility remains a significant challenge due to polydispersity and variability in bio-capping layers across batches [128]. The presence of numerous uncharacterized biomolecules in plant crude extracts, along with poor reproducibility among the experimental runs, should be highlighted in addition to considering the lack of structural characterization of the organic shell in the green synthesis literature.

Qayyum et al. [129] confirmed that the AgNP interaction with bacterial cells resulted in ROS generation, subsequent cellular membrane damage, bacterial cell lysis, and ultimate cell death. Silver NPs were greenly synthesized using a *Mangifera indica* aqueous extract and tested for antibacterial activity through MIC value determinations. The bacteria-suppressing ability of the resulting AgNPs against both Gram-negative (*Klebsiella pneumoniae*, *Pseudomonas aeruginosa* and *Escherichia coli*) and Gram-positive (*Streptococcus mutans* and *Staphylococcus aureus*) pathogens was high even compared to chemically obtained AgNPs. The MIC values were 8 mg/L and 16 mg/L for the two groups of strains tested, respectively. Leakage of proteins and reducing sugars from the AgNP-treated cells confirmed cell membrane deterioration. Furthermore, the researchers [129] conducted cellular ROS detection assays via an analytical technique. The outcomes revealed that nanomaterial-produced ROS were generated upon AgNP contact with bacterial cells in a time-dependent manner, followed by experimentally confirmed damage to cell walls and DNA, thus suggesting a distorted bacterial biofilm structure caused by AgNPs.

Today, many researchers have come to understand that AgNPs combined with phytochemicals are a powerful weapon against multidrug-resistant pathogenic bacteria and that bioreducers and biostabilizers may act as suitable and inexpensive alternatives to hazardous chemicals for the fabrication of AgNPs. Quercetin and yellow pepper extract, known to contain quercetin in the highest amount compared to other flavonoids, were used separately to obtain biofabricated AgNPs [130]. The antibacterial action against extended-spectrum β-lactamase-producing *Pseudomonas aeruginosa* and *Escherichia coli*, as well as methicillin-resistant *Staphylococcus aureus*, was revealed to be based on an increased production of ROS and bacterial membrane permeability. Liao et al. [131] used branched cyclodextrin to synthesize AgNPs via stable complex formation and reduced NP agglomeration. Transmission electron microscopy showed that the resulting AgNPs could enter the multidrug-resistant bacteria *Pseudomonas aeruginosa* and impair its morphology and structure, in combination with ROS production at the early stage of AgNP interaction with the bacteria. Bao et al. [132] confirmed the antibacterial effect of AgNPs on *Escherichia coli* and concluded that AgNPs can simultaneously induce apoptosis and inhibit new DNA synthesis in cells in a positive concentration-dependent manner.

### 4.2. Copper–Phytochemical Antibacterial Nanocompositions

Cu-based NPs may represent a promising antibacterial agent, provided that additional examinations are conducted to confirm their safety profile. Copper-based NPs (CuO and CuNPs) are among the most common metal NPs from an applicability viewpoint, e.g., catalytic activity promotion and inhibition of pathogenic bacteria [133], with moderate values of minimal bactericidal concentrations (100 µg/mL or higher). However, the observed phytotoxic properties of CuONPs should be taken into account. For instance, CuONPs influenced both transgenic and conventional cotton via the inhibition of growth and developmental parameters and phytohormone content [133]. Cu-based NPs, especially cuprous and cupric oxides (Cu_2_O and CuO, respectively) in their nano-form, have aroused particular interest in antibacterial material development for elimination of various pathogenic microbes, ranging from water purification to wound sterilization [134]. Multitoxicity renders copper effective against drug-resistant bacteria like methicillin-resistant *Staphylococcus aureus* [135] and carbapenemase-resistant bacteria [16]. These properties of CuONPs may be beneficial for their biomedical applications, such as tailored equipment with biocidal effects, modified textiles, and so on [136]. Many studies have revealed that these NPs have excellent antibacterial activity against several human pathogens, including methicillin-resistant *Staphylococcus aureus* strains [137]. For instance, a very recent work by Yuan and Wang [138] used a rose (*Rosa centifolia*) petal extract to synthesize CuNPs, followed by an evaluation of the antibacterial efficiency of the prepared NPs in inhibiting the growth of a multidrug-resistant strain of *Escherichia coli*. However, the cytotoxicity observed with mouse macrophage J774 cells appeared to be greater compared to three standard antibiotics tested.

The toxicity of Cu-containing NPs is mainly explained by ROS-induced generation [139]. ROS production is currently recognized as a principal antibacterial mechanism that occurs when CuONPs are capable of generating superoxide and hydroxyl radicals [140]. Several other researchers also reported that ROS formation inside bacterial cells was elevated under the influence of CuO aqueous suspensions [141]. ROS induced by CuONPs damage the cell membrane and proton efflux pumps, ultimately resulting in the death of harmful bacteria [142]. Closely linked to increased ROS generation, DNA damage and lowered GSH (reduced glutathione) content compared to background levels may also contribute to the higher toxicity of CuONPs compared to bulk CuO and cationic Cu^2+^ chemical forms [143,144].

The synthesis of CuONPs using *Phyllanthus amarus* leaf extract was performed [30]. The assessed NP antimicrobial activity manifested a considerable inhibition of both the Gram-negative (*Pseudomonas aeruginosa* and *Escherichia coli*) and Gram-positive (*Staphylococcus aureus* and *Bacillus subtilis*) strains implemented in the research. The effect of the CuONPs was as high as 155% of that of the positive control, antibiotic rifampicin. The MIC values determined for the phytosynthesized CuONPs were improved by 22% of the rifampicin value versus Gram-negative bacteria and by 32% of the positive control value versus Gram-positive bacteria.

Copper-based NPs can act as Fenton-active catalysts that work over a broader pH range as compared to the Fe-based process [14]. Cuprous oxide-based nanocomposites are especially suitable Fenton-like catalysts for wide applications, leading to the innovative preparation of bimetallic nanostructures. These nanomaterials facilitate Fenton-like reactions in a hydrogen peroxide self-feeding manner [145] to yield highly improved bacterial inactivation. CuNPs produce ROS in bacteria and fungi, thus providing antibacterial and antifungal properties. CuNPs have been shown to display anticancer properties. Above the optimal concentration, these NPs are harmful to animals and affect several organs in human beings. Copper and zinc oxide-based NPs are well documented as pathogenic bacteria inhibitors.

### 4.3. Metal Oxide–Phytochemical Antibacterial Nanocompositions

Many researchers discuss metal oxide-containing NPs as smart NPs for antimicrobial activity. Titanium(IV) oxide NPs (TiO_2_NPs) possess chemical resistance, remain stable in both alkaline and acidic conditions, are low toxic [146] and are widely used as catalysts in cosmetics, sunscreens and plastics owing to their catalytic, optical, and antibacterial properties [147]. Owing to their capability of destroying bacterial outer membranes in the presence of light, these nanostructures are implemented in antimicrobial photocatalytic technology directly linked to the ROS-mediated pathway. A nanostructured formulation consisting of titanium dioxide and zinc (TiO_2_/Zn) caused a significant reduction in the survival of the bacterial pathogen *Xanthomonas* sp., a causative agent of chlorosis and necrotic spots on leaves that is tolerant to Cu-containing bactericides [15]. In the presence of light, TiO_2_NPs create extremely reactive hydroxyl and superoxide free radicals that are detrimental to target cells. Importantly, bulk counterparts of TiO_2_NPs and ZnONPs, i.e., TiO_2_ and ZnO, displayed no antibacterial activity against *Dickeya dadantii*, a causative agent of sweet potato stem and root rot disease [34]. In contrast, both nanomaterials, TiO_2_NPs and ZnONPs, readily inhibited (at a low level of 50 µg/mL) *D. dadantii*, including bacterial biofilm formation.

Non-noble metals and biometal-based NPs, as constituents of antibacterial compositions, are especially attractive for biotechnological applications owing to their environmentally friendly nature and safety for human health. Zinc-based NPs have gained wide biomedical applications as nanomaterials featuring low toxicity, biocompatibility, and profound antibacterial properties attributed to zinc cation release, ROS generation, and apoptosis induction in bacterial cells [148]. ZnO- and MgO-containing NPs were examined for their biocidal efficiencies and cell wall interactions [32]. The underlying antibacterial mechanism of all three oxide-type NPs was related to ROS generation. The properties of nanostructured MgO open vast opportunities for developing MgONP composites as promising antibacterial agents [149]. MgO-based nanomaterials are particularly interesting because of their high thermal stability, low cost, environmental compatibility and considerable application potential in food safety [150]. However, research on the action of MgONPs against several important foodborne pathogens, including *Campylobacter jejuni*, *Escherichia coli* O157:H7, and *Salmonella enteritidis*, has revealed high MIC values. To completely inactivate *C. jejuni* at 10^8−9^ CFU/mL, a minimal concentration of 2 mg/mL MgONPs was required, while *E. coli* O157:H7 and *S. enteritidis* required at least 8 mg/mL MgONPs [150]. In contrast, phytofunctionalized MgONPs showed a wide spectrum of bactericidal activity against both Gram-positive and Gram-negative bacteria, the capability of enhancing cytosolic ROS, and membranolytic properties confirmed with model cell membranes [151].

Numerous works reported the antibacterial properties of zinc oxide NPs, ZnONPs, with respect to a wide spectrum of pathogens [152]. When identifying the mechanisms behind ZnONPs’ action on two *Bacillus* species, *Bacillus thuringiensis* and *Bacillus megaterium*, inhibited cell growth and stimulated endospore formation were noted. ZnONP implementation caused oxidative stress, detected by changes in superoxide anion production in *Bacillus* strains and quantified by means of superoxide dismutase enzymatic activity measurements [35].

In general, ZnONPs were found to be efficient in suppressing both Gram-positive and Gram-negative bacteria [36]. Phytosynthesis of ZnONPs using a *Phyllanthus embilica* stem extract and subsequent examination of antibacterial traits demonstrated a remarkable antagonistic activity of ZnONPs against the pathogens *Salmonella typhi* and *Klebsiella phnemonea*, evidenced by ROS production initiating bacteria eradication [36]. The preparation of ZnONPs using an aqueous extract of *Salvadora persica* twigs/bark (Miswak) was attempted [153]. The phytochemicals of *S. persica* (phenolic acids, flavonoids, alkaloids, saponins) enhance NP stability and biological activity. The synthesized Zn-containing nano-phase was monodispersed, consisting of 21 nm sized spheres of ZnONPs, displaying considerable stability with a zeta potential of −41 mV and convincing antimicrobial activity against *Staphylococcus aureus*. Miswak-mediated ZnONPs exhibited superior antioxidant potential (82.15%) and cytotoxic potency (LC_50_ of 0.81 µg/mL), low hemolysis, and compatibility with human red blood cells. Due to the released metal ions and catalyzed redox reactions, the ZnONPs elevated bacterial intracellular ROS, followed by disruption of cellular redox homeostasis and subsequent oxidative damage to bacterial macromolecules. The pronounced antibacterial potential displayed by ZnONPs suggests that the phytochemical matrix of *Salvadora persica* may provide an additional synergistic mechanism.

Titanium dioxide NPs produced using the 3,4,5,7-tetrahydroxy-flavone luteolin (Figure 2) also induced intracellular ROS generation and caused mitochondrial disruption in cells, leading to intrinsic apoptosis [154]. Maheswari et al. studied the bioproduction of TiO_2_NPs assisted with *Plectranthus amboinicus*, *Phyllanthus niruri*, and *Euphorbia hirta* [33]. The spherically shaped TiO_2_NPs were 4 nm to 15 nm in diameter and exhibited moderate antibacterial properties with respect to *Pseudomonas aeruginosa*, *Klebsiella pneumonia*, *Escherichia coli*, and *Staphylococcus aureus*, along with excellent effects against *Streptococcus mutans*, which could imply a future pharmaceutical application of phytochemical-modified TiO_2_NPs.

The data implicate common mechanisms of toxicity induction in bacteria treated with the plant-derived TiO_2_NPs and ZnONPs. The involved pathways are related to the internalization and uniform distribution of nanomaterials in microorganisms, ROS production, low-molecular-mass cellular antioxidant depletion accompanied by increased lipid peroxidation, and oxidative DNA injury leading to bacterial cell death [148,155].

### 4.4. Nano-Fe–Phytochemical Antibacterial Nanocompositions

A decade ago, there was collaborative work on the antibacterial activity of iron oxide nanoparticles (IONPs) [156]. Rapid synthesis of crystalline Fe_3_O_4_NPs comprising iron in mixed oxidation states Fe(+2) and Fe(+3) was performed by reduction of ferric chloride (FeCl_3_) with an aqueous leaf extract of *Tridax procumbens* [157]. The reduction of Fe(+3) to Fe_3_O_4_NPs was presumably due to the carbohydrates in the *T. procumbens* extract. The antibacterial effect of these phytosynthesized IONPs was detected against Gram-negative bacteria, including *Pseudomonas aeruginosa*. Fe_3_O_4_NPs were shown to be an effective bactericide.

Zero-valent iron nanoparticle (Fe^0^NP) solutions comprise different oxidized forms of iron. It is believed that the physicochemical properties of Fe^0^NPs, such as chemical reactivity, particle aggregation, etc., change in an aqueous medium when Fe^0^ is oxidized to its oxides. This inevitably influences the bioavailability, uptake, and toxicity behavior of Fe-based NPs [158]. Capping Fe^0^NPs with some plant molecules may prevent this oxidation. The reduction of iron salt (ferric chloride) to Fe^0^NPs was observed as a color change from yellow to greenish-black at room temperature. The synthesized nanoparticles showed a spherical morphology and an average size of 27 nm. Antibacterial activity of phytosynthesized NPs assayed against human pathogens, namely, Gram-negative bacteria *Escherichia coli*, *Klebsiella pneumonia*, *Pseudomonas fluorescens* and Gram-positive bacteria *Staphylococcus aureus* and *Bacillus subtilis*, was judged to be satisfactorily good [156,157]. Iron–polyphenol NPs (Fe-Phen NPs) were obtained using leaf extracts of Australian native plants, including *Melaleuca nesophila*, *Rosmarinus officinalis*, and *Eucalyptus tereticornis*. Fe cations interact with polyphenols to form composite-type amorphous NPs with sizes of 50 to 90 nm. The proposed chemical structure of Fe-Phen NPs contained Fe^2+^ ions coordinated with three bidentate ligands based on ortho-diphenol (catechol) derivatives [159]. The resulting iron complex NPs, or chelated ferric–polyphenol NPs, are effective heterogeneous Fenton catalysts participating in Fenton-like reactions with Fe-Phen NPs.

Further research has valued IONPs for their magnetic properties and more appropriate toxicity and biocompatibility compared to other metal NPs. Some studies indicate that Fe_2_O_3_NPs influence both Gram-positive and Gram-negative bacteria similarly, while others report specific effects on Gram-negative bacteria, which can be attributed to variations in their cell wall structures and metabolic processes [160]. Dharshini et al. demonstrated the biosynthesis of Fe^0^NPs using a *Phyllanthus reticulatus* (Black honey shrub) leaf extract [161]. The reduction of ferrous sulphate Fe(+2) to Fe^0^ was confirmed by the color change of a solution from yellow to black. The shape, Fe(0) oxidation state and crystalline nature of Fe^0^NPs were proved by SEM, EDX, and X-ray diffraction analyses. The particle size range of phytosynthesized Fe^0^NPs was determined using a particle size analyzer. The NPs had an irregular spherical shape and an average size of 185.6 nm. Antibacterial activity measured in the bacterium growth inhibition zone indicated the significant antagonistic potential of these NPs against a wide range of both Gram-positive and Gram-negative bacteria, resulting in ROS-mediated bacterial cell death.

Plant-mediated synthesis of IONPs using leaf extract of *Rhamnus virgata* Roxb. as a potential stabilizing and reducing agent is reported [162]. The bioreduction of precursor salts by the extract yielded a hematite phase of α-Fe_2_O_3_. As measured through dynamic light scattering (DLS) analysis, the average size of the IONPs was about 20 nm, and there were also particle agglomerates up to 174 nm. The shape of the IONPs appeared to be spherical. The elemental composition of IONPs, studied using EDS, showed that only iron and oxygen were present in the sample, confirming the pure crystalline nature of IONPs. Diverse biological activity, including antibacterial activity, was also evaluated. The bacterial strains most susceptible to the phytosynthesized IONPs were *Bacillus subtilis* and *Escherichia coli*, both strains characterized by MIC values of 31.25 μg/mL, while the least susceptible pathogens were *Klebsiella pneumonia* and *Pseudomonas aeruginosa*, with MIC values of 125 μg/mL. The authors [162] again relate the antibacterial potential of phytosynthesized IONPs to ROS generation as the key reason for oxidative damage to bacterial cells. They also consider the significance of the bioactive components of *R. virgata* leaf extract in capping and stabilizing Fe-based NPs and thus in the observed antibacterial potential. Very recently, much smaller-sized IONPs, i.e., Fe_2_O_3_NPs with an average diameter of 15 nm, were bioproduced through a *Thymus migricus* extract [160]. The antibacterial activity of the Fe_2_O_3_NPs was assessed using the disc diffusion technique against one Gram-positive bacterial strain (*Staphylococcus aureus*) and three Gram-negative bacterial strains (*Escherichia coli*, *Pseudomonas syringae*, and *Salmonella enterica*). Appreciable bacterial growth inhibition was observed for *S. aureus* and *S. enterica*; however, a high concentration of 400 μg/mL Fe_2_O_3_NPs was required. In general, the previous studies demonstrate the decisive role played by plant secondary metabolites in the phytosynthesis of iron-based NPs, specifically in striking a compromise between particle size and antibacterial efficiency of the resulting nanocompositions.

## 5. Specific Mechanisms Behind Antibacterial Strategies Based on ROS in Metal NPs: Possible Players and Inherent Limitations

### 5.1. How Organic Capping Layers Genuinely Influence Synergistic Mechanisms in the Metal NP System

The idea to create sophisticated metal–organic interfaces under ambient conditions using plant metabolites seems attractive. Phytochemical engineering of metal NPs leads to the enhanced bacteriostatic or bactericidal ability of this biomodified metal nanomaterial. Biologically derived systems are associated with both advantages and challenges. Compared to chemically synthesized analogues, biogenic NPs consistently exhibit greater structural heterogeneity and organic–inorganic interfacial complexity, which can enhance antibacterial performance but also introduce variability in reproducibility.

General plant extract bioactivity should not be confused with genuine nanohybrid synergistic mechanisms. The surface characteristics of organic-covered NPs directly influence catalytic activity, selectivity, and stability by modulating adsorption behavior and charge transfer processes [38]. The number of works elucidating how organic capping layers genuinely influence interfacial charge transfer is rather limited. Metal NPs have been repeatedly referred to as nanohybrid materials that promote intracellular ROS accumulation in bacteria by disturbing the electron transfer process. The antibacterial effects of biocapped NPs are also based in part on electron-transfer-induced ROS accumulation [163]. In this way, NPs can produce chemical conditions that induce a prooxidant environment in bacterial cells. Furthermore, the metallic core’s synergistic interaction with biological ligands optimizes adsorption–desorption energetics [104]. Bioorganic ligands can either modify the electron density of the NP metal core or physically block specific steric locations of a reactant molecule, strategically directing reaction routes.

Plant metabolites implemented in metal NP biofabrication are capable of providing a strong conductive support in the form of organic capping layers that allow for fast electron transfer [164]. Quantitative measures show that metal NPs, as biogenic systems, use efficient electron transport pathways [165]. The biocapping layer prevents metal particles from detaching or metal cations from excessive leaching during operation performance [53], thus influencing ion dissolution kinetics. Biogenic particles are thought to have surface flaws and undercoordinated sites, which could lead to enhanced crystal defect densities [106]. Spatial confinement caused by the organic capping layers in NPs prevents particles from sticking to one another or sintering during overheating or mechanical stresses [53]. This confinement effect provides a mechanistic basis for understanding NP nucleation and growth, but it also brings up an issue of how organic capping layers genuinely influence catalytic activity.

Catalytic activation happens by chemisorption, in which reactant molecules form chemical bonds with metallic centers, weakening internal molecular bonds. By means of metal NP capping with organic material, catalytic NPs may be prepared with tunable particle sizes, shapes, and compositions. NPs synthesized with different organic capping layers display modulated catalytic activity owing to chemical fingerprints of the stabilizing ligands and surface functionalization provided by plant metabolites. Phytolayers can enhance colloidal stability and chemoselectivity, but excessive surface ligands may obstruct active sites and reduce overall catalytic turnover. The adsorption must be strong enough to activate the reactant but weak enough to allow the product to exit without contaminating the environment [52]. Some portion of the capping layer blocks the reactive sites and affects the catalytic turnover rate. Concurrently, in the course of many chemical reactions, the reactant tends to block reactive sites, causing catalytic deactivation; consequently, a balanced manufacturing approach accompanied by standardized benchmarking frameworks is necessary.

In photocatalysis, the key engineering problem is the quick recombination of electron–hole pairs. Bioengineered devices overcome this issue by utilizing the doping action of biological heteroatoms. Nitrogen and sulphur atoms form mid-gap states that trap photogenerated carriers, enhancing their lifetimes and the likelihood of a surface redox reaction [106]. The organic capping layer can promote substrate adsorption through π–π interactions or hydrogen bonding, depending on the molecular structure of the reactant. This ensures that photogenerated radicals are produced in the immediate vicinity of the substrate [53]. This spatial proximity optimizes the quantum yield and overall rate constant of the photocatalytic process, offering a roadmap for the creation of next-generation green photocatalysts. Very recent studies highlight a significant impact of nanoscale defect engineering, active site density, and surface–biomolecule interactions on catalytic performance [165]. UV-ozone treatment aids in partially removing organic adsorbates from metal NP surfaces, and the results imply that the nature of chemical bonding between organic capping layers and NP surfaces plays an important, if not determining, role in the bioactivity of metal particulate systems. However, the field remains constrained by significant variability in reported performance metrics and a lack of standardized evaluation protocols. Differences in synthesis conditions, catalyst composition, and testing methodologies often lead to inconsistencies that complicate direct comparison with conventional synthetic catalysts.

### 5.2. Oxidative Stress in Bacteria

**Figure 3:**
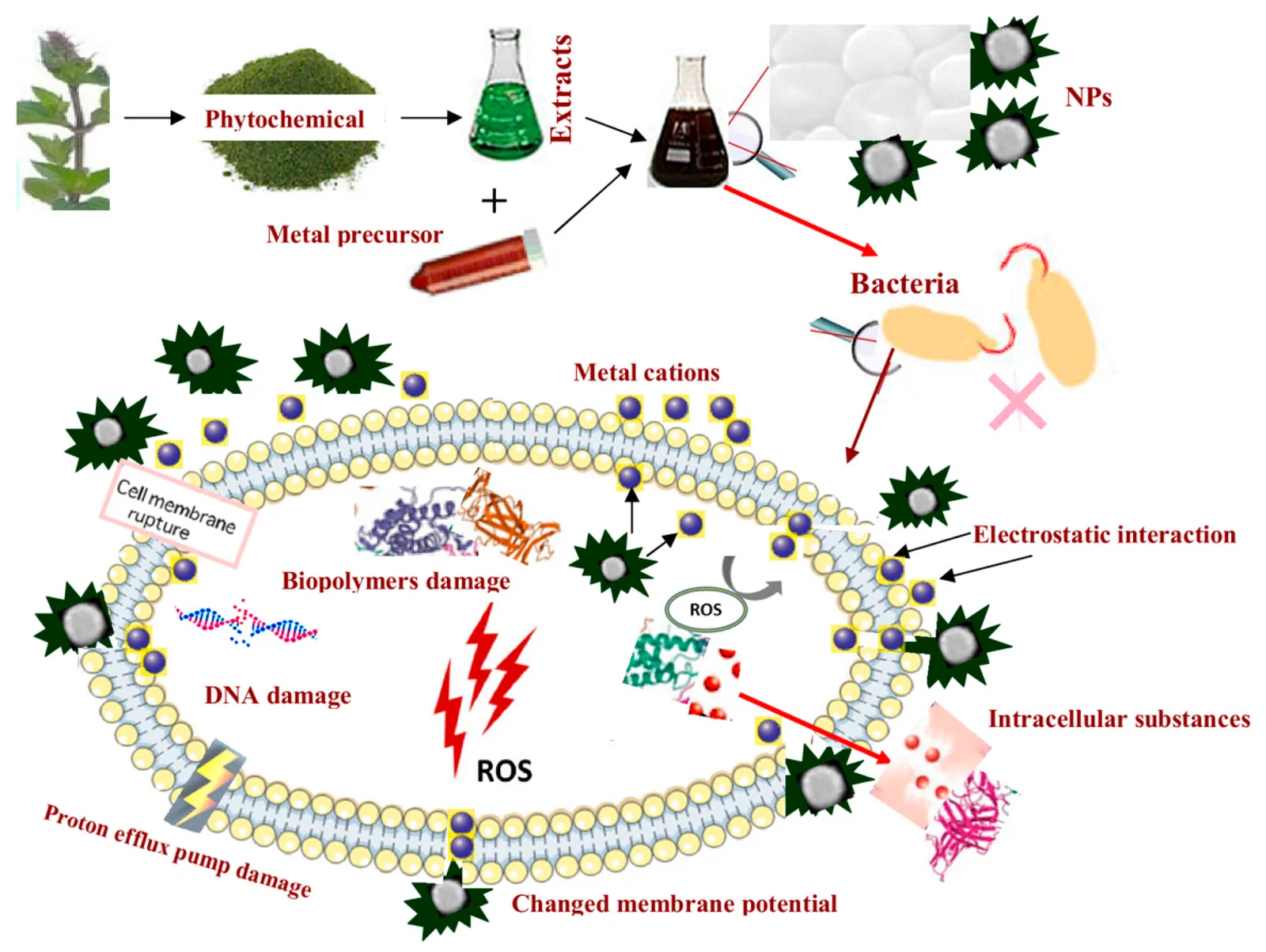
Schematic representation of phytosynthesis and the ROS-mediated antibacterial mechanism of metal NPs.

Oxidative stress is recognized as one of the principal mechanisms by which metal NPs induce lethal effects in bacterial cells [100]. The main pathways rely on a disequilibrium of oxidation and antioxidation processes and the failure of bacteria to recover from the injuring action of overproduced ROS, manifested as lipid peroxidation, membrane permeability augmentation, and oxidative damage to DNA, RNA, and proteins. NPs interact with redox-active enzymes like NADPH oxidase and disrupt the mitochondrial electron transport chain. Bacterial oxidative phosphorylation processes appear to be impaired, and ATP generation attenuated; accordingly, the distorted metabolism in bacteria facilitates ultimate cell death [163]. Metal NPs can initially be absorbed on the surface of the cell and then undermine the cell membrane; they may be transported into the cytoplasm, thus exposing them to a variety of biomolecules, which is followed by DNA aggregation, protein degradation, and intracellular substance leakage (Figure 3).

The greater attention of several authors to nanomaterials is focused on overcoming bacterial resistance mechanisms through mechanical damage to cells [166]. This disruption occurs via interactions between NPs and bacterial cell walls, leading to compromised bacterial cell integrity. The term “nano-antibiotic mechanism” [167,168] means disorganization of the bacterial membrane resulting from contact with unwanted or foreign objects, such as common antibiotics or metal NPs. This can cause a malfunction of the permeability barrier, followed by a loss of membrane integrity and the penetration of an unfavorable substance or object. Higher surface energy due to a greater specific surface area favors the adsorption of metal NPs onto bacteria, which appear to be deprived of the exchange of matter and energy with the environment, and so of normal metabolism.

### 5.3. Photocatalytic Antibacterial Mechanism

A few types of ROS, namely, superoxide anion radicals (•O_2_^−^), hydroxyl radicals (•OH), and singlet oxygen (^1^O_2_), contribute to the majority of the oxidative stress-induced processes in biological systems. Photocatalytic reactions of some metal oxide-based materials generate all these types of ROS, serving as the main mediators of photocytoxicity and thus providing antibacterial effects.

**Figure 4:**
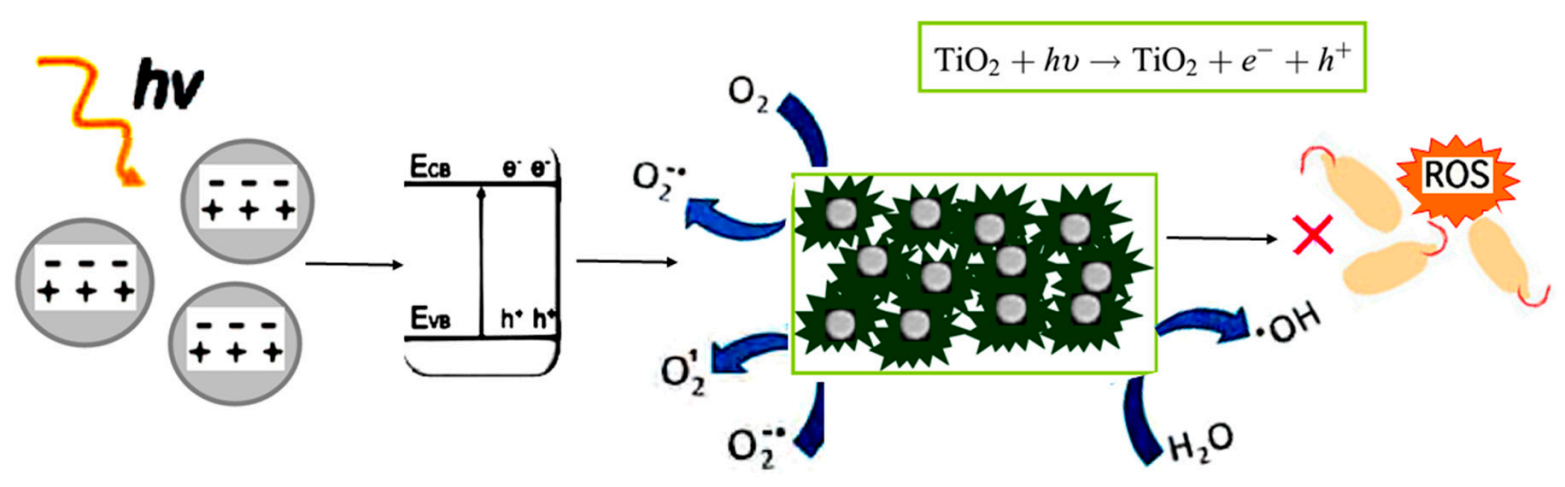
Simplified representation of titania irradiation with light to induce electron transfer and leave holes.

The photocatalytic properties of titania (TiO_2_) are well known. The antimicrobial activity of photoactivated TiO_2_ was first demonstrated about forty years ago, and since then, multiple studies have evidenced that photocatalysis is capable of killing a wide range of Gram-negative and Gram-positive bacteria [169]. TiO_2_ is a wide-band semiconductor. The band gap energy (energy required to promote an electron) of anatase, the most effective photocatalyst among the polymorphs of TiO_2_, is about 3.2 eV [170]. Through TiO_2_ irradiation with light whose energy is equal to or higher than the band gap energy of TiO_2_, electrons transfer from the valence band to the conduction band, and positively charged holes are generated in the valence band (Figure 4).

Both electrons and holes are mobile owing to electron migration within the conduction band and the efficient hole neutralization by the extra free electron from an adjacent molecule [171]. The electron and hole bulk recombination process was identified to be the ROS-producing reaction when the electrons and holes reach the surface. In solution, these can react to give hydrogen peroxide, hydroxyl and hydroperoxyl radicals. Valence band holes are powerful oxidants, while conduction band electrons are good reductants. So, molecules that contact a photocatalyst can be oxidized or reduced, and the biomolecules of bacterial pathogens can be ultimately degraded via contact with photoactivated TiO_2_. Additional factors preventing the electrons and holes from recombination, thus promoting the subsequent redox reactions, are found to be efficient scavengers or surface defect states [172].

In addition, there is a need to transfer the absorption of titania to the more practical visible spectral range, which seems achievable through the formation of interfacial charge transfer complexes with small organic molecules [173]. The recombination rate of the electron–hole pairs could be reduced, and the interfacial charge transfer efficiency increased, by titania NP surface treatments, including modification by phytochemicals to yield novel visible-light-responsive complexes between TiO_2_ and plant secondary metabolites. This area is currently the subject of intensive research.

Zinc oxide is a multifunctional semiconductor material [168]. Its unique characteristics endow nanostructured ZnO-based materials with excellent catalytic activity, biocompatibility and novel biological functionalities. In particular, the promising antibacterial activity of ZnO nanostructures has attracted the attention of biomaterial researchers. ROS generated by the photocatalytic reaction of ZnO typically include hydroxyl radicals, singlet oxygen and superoxide radicals; all are toxic to bacteria because they damage cellular macromolecules. Consequently, the generation of ROS has been recognized as the predominant antimicrobial mechanism underlying the effects of photoexcited ZnO on bacterial cells. Of importance also are the patterns linked to the impairment of bacterial membrane integrity upon contact with specific ZnO nanostructures, such as nanoneedles and quercetin-loaded ZnO nanorods [174]. The enhancement in bacterial photocatalytic inactivation by the synthesized flower-like ZnO micro/nanostructures [175] may be attributed to this unique morphology, which can increase the amount of surface hydroxyl groups and the quantity of photogenerated electrons and holes available to participate in the photocatalytic reaction.

The electron reacts with molecular oxygen (O_2_) through a reductive process to generate superoxide anion radicals (•O_2_^−^). The hole can capture the electron from water and/or hydroxyl ions to generate hydroxyl radicals (•OH) through an oxidative process. Singlet oxygen (^1^O_2_) is mostly produced indirectly from •O_2_^−^ reactions in solution. Recombination of two •OH radicals produces hydrogen peroxide (H_2_O_2_). Some researchers suggested that the chemical interactions between bacteria and hydrogen peroxide generated from metal NPs were the predominant mechanism behind the antibacterial properties of NPs. However, the level of H_2_O_2_ detected in ZnONP and MgONP suspensions was found to be relatively low [150], so it is possible that the production of H_2_O_2_ from nanoparticles is not the dominating mechanism underlying the antibacterial activity of these metal oxide NPs. Furthermore, ZnO suspensions with different particle sizes have different disinfection mechanisms against bacterial pathogens, with smaller aggregates (less than 100 nm) showing better antibacterial activity with no hydrogen peroxide detected in their suspensions [176]. Much more likely, the generated H_2_O_2_ contributes to bactericidal activity by inducing oxidative stress in cells following the antibacterial treatment [177].

Consequently, according to the available research, the antibacterial characteristics of semiconducting nanocrystals are primarily attributed to their ability to generate ROS upon irradiation with UV or visible light [178]. ROS production can be triggered by electron/hole pairs or crystal defects, such as oxygen vacancies, as well as enhanced interstitial zinc within NPs. The latter’s surface is a trap for charge carriers and the centers of their nonradiative recombination, whereas crystal defects serve as trap centers by capturing and storing electrons or holes during photoexcitation, thus governing the dynamics of these charge carriers [179]. Compared to the chemical method of NP synthesis, nanostructured metal NPs prepared using plant extracts are characterized by the presence of excessive amounts of defects, a decrease in optical band gap owing to these defects, and thus enhanced photocatalytic efficiency [180]. These NPs directly engage with bacterial biomolecules like lipid membranes, inducing the generation of superoxide (•O_2_^−^) radicals.

### 5.4. Ion-Release Mediated Antibacterial Mechanisms

**Figure 5:**
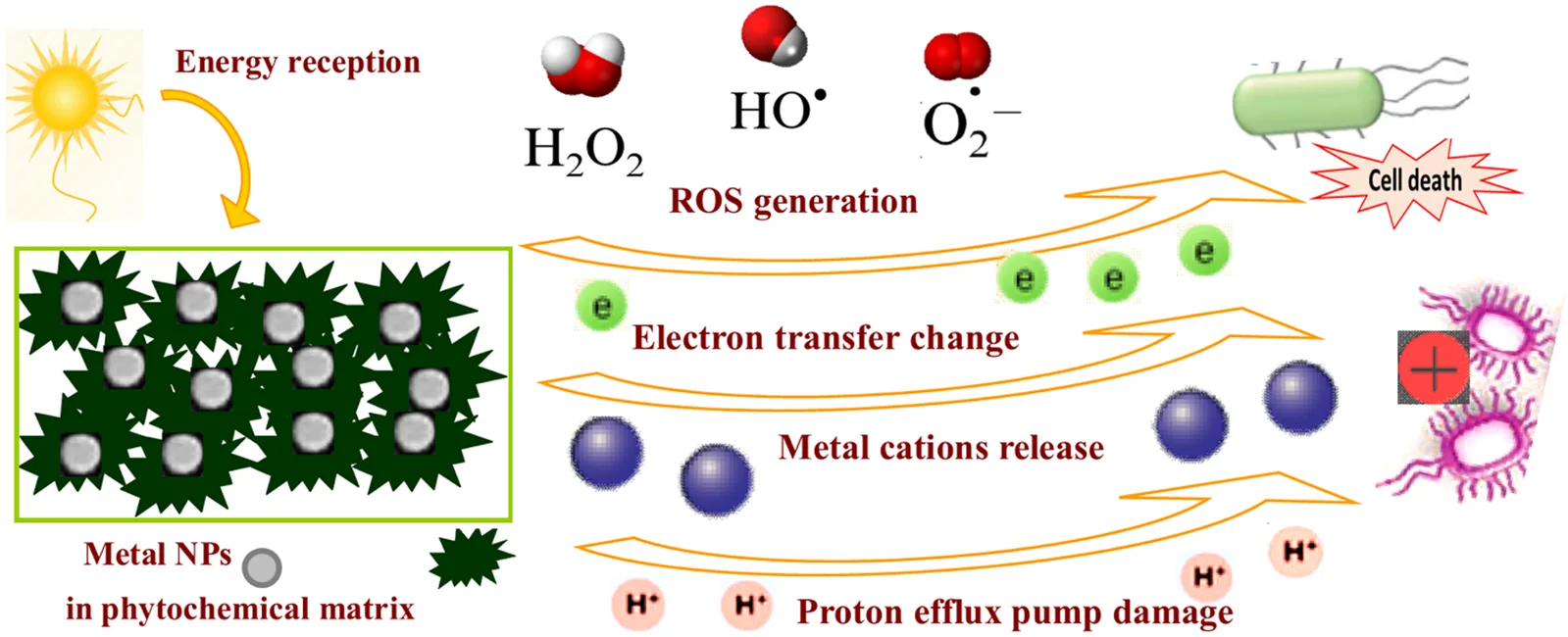
Illustration of a metal-containing nanomaterial’s antibacterial toxicity based on different mechanisms.

The rate at which a photocatalytic reaction occurs is influenced by the presence of metal ions in a metal NP-based material, which leads to a decrease in recombination processes, an improvement in carrier lifetimes, and an increase in photocatalytic activity. Therefore, cation release from metal NPs is directly related to the material’s antibacterial properties (Figure 5).

An interaction between NPs and a solvent (more frequently, a water-based one) leads to solvation processes and subsequent release of toxic ions, disrupting a biological system’s normal functions. Several studies have implied that the toxicity of metal NPs is primarily attributed to NP dissolution and the consequent ROS production. Metal cations emitted during dissolution interact with cellular organelles, which subsequently leads to mitochondrial membrane potential loss, mitogen-activated protein kinase pathway activation, and finally, bacterial cell apoptosis [181].

In particular, the release of zinc cations from ZnONPs can ultimately lead to fatal damage to the bacterial cell membrane [167]. Part of the Zn^2+^ ions released from ZnO nanostructures adsorbs onto the bacterial surface through electrostatic force. Interactions between cations and bacteria can disrupt the charge balance, leading to severe cell distortion until bacterial death due to lytic processes. Additionally, ROS production interrupts the redox balance within cells, resulting in oxidative stress. The •O_2_^−^ radicals produced by ZnONPs possess high potency in damaging bacterial biomolecules [178].

Furthermore, Zn^2+^ ions penetrate the cell membrane and react with some molecular moieties, such as sulfhydryl groups (-SH), amino groups (-NH_2_), hydroxyl groups (-OH), etc., on biopolymers, altering their structure and functioning, which induces unbalanced bacterial metabolism and death. Zinc cations have a special affinity to bacterial sulfhydryl groups. An examination of Zn-containing protein structures deposited in the Protein Data Bank has again supported the affinity between S–H coordinating motifs and Zn, whose most frequently identified ligand groups were revealed to be Cys_4_, Cys_3_His, and Cys_2_His_2_ [182]. Consequently, Zn cations can bind thiol groups to inhibit the action of bacterial respiratory enzymes and eventually lead to cell death.

Zinc cation interference with the electron transport chain exacerbates ROS production. The release of copper cations from destabilized NPs, both Cu^2+^ and Cu^+^ ions, causes ROS production, lipid peroxidation, decreased ATP content, and DNA and protein degradation because of oxidation and Fe replacement by Cu in proteins with Fe–S moieties [183,184]. Cations released by metal nanomaterials enter bacteria in different ways. For Gram-positive bacteria possessing a much thicker peptidoglycan layer than Gram-negative bacteria, the cell wall features a complex composition consisting of teichoic acid and lipoteichoic acid, so metal ions can be coordinated by these acids and transported into a cell. For Gram-negative bacteria, with an additional outer membrane composed of lipopolysaccharides (LPSs) and phospholipids, selective permeability is further reinforced by porins, which allow only small hydrophilic compounds to pass through and act as ion channels to promote passive diffusion into cells. The negative charge on LPSs allows their electrostatic interactions with metal-based NPs and can disrupt bacterial membrane integrity [185].

A significant increase in the bacterial intracellular concentration of silver ions may be caused by AgNPs’ ability to effectively bind to the bacterial membrane and rupture its structure. The authors of one study reached the conclusion that nanostructured silver certainly possesses an additional bactericidal factor compared to silver cations [186]. The highest bactericidal effect of sphere-shaped AgNPs, as compared to silver nanorods, nanotriangles and nanohexagons, against Gram-positive and Gram-negative bacteria was attributed to the heaviest release of Ag^+^ ions over NPs of other shapes [187].

When discussing cation release from metal NPs, the process of counterpart electron transfer should be noted. The antibacterial activity of NPs increases with oxygen vacancy concentration growth since the surface-enriched electron concentration provides a more reactive site for activating ROS production [188]. Electron transfer processes generate active electrons that affect the bacterial surface, leading to subsequent biochemical reactions that inactivate the respiratory chain, induce the formation of harmful radicals, and result in bacterial cell inactivation.

Inhibition of respiratory enzyme function due to free radical formation, and inactivation of several enzymes comprising thiol groups in their structures are among the mechanisms underlying the antibacterial action of AgNPs [94]. Liao et al. confirmed that although AgNPs induced ROS improvement, the superoxide anion radical content did not markedly increase in multidrug-resistant *Pseudomonas aeruginosa* [131]. It was demonstrated that silver ions inactivate enzymes by binding sulfhydryl groups in proteins and promoting the release of iron, with subsequent hydroxyl radical formation by an indirect mechanism likely mediated by ROS [189].

There is still some debate regarding the antibacterial mechanism of Zn^2+^ ions. Li et al. [47] evaluated the effect of zinc ion release on antibacterial activity by monitoring the release of zinc cations from suspensions of different ZnONPs. A common antibacterial assay with *E. coli* cells was conducted. The results showed no significant inhibitory effect toward bacteria even when the concentration of Zn^2+^ was as high as 1 mg/L. This finding implied a minor antibacterial effect exerted by the released zinc cations. The principal antibacterial mechanisms of ZnONPs may be different in different media, or depend on NPs’ distinct physicochemical properties, and dissolved Zn-containing charged species can be altered by the medium components. The detailed mechanism underlying the antimicrobial activity of ZnO is still under debate and requires further investigation [167].

### 5.5. Mechanism Mediated by Fenton-like Oxidation Systems

**Figure 6:**
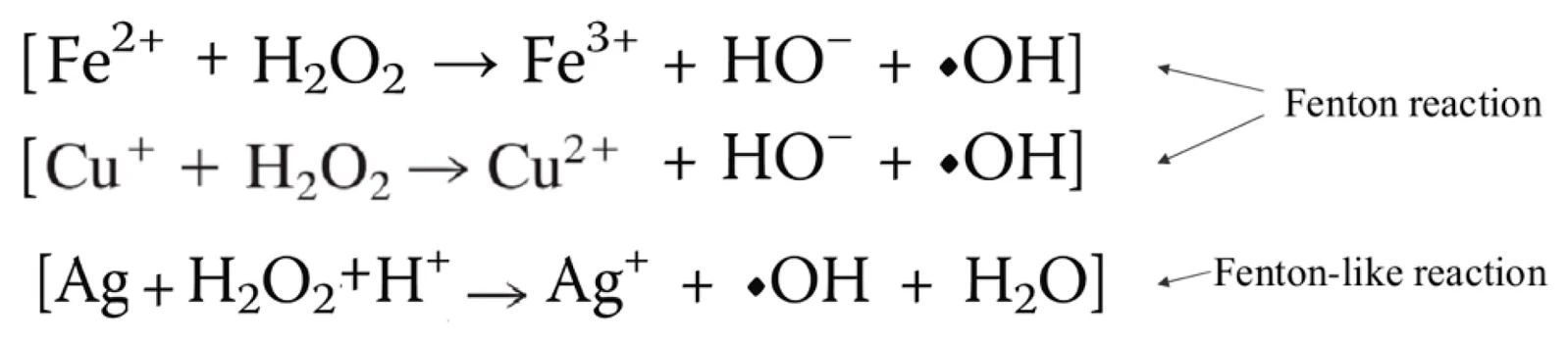
Schematic representation of Fenton-like reactions.

ROS bursts alter the normal physiological functions of cells. The most supported explanation for the cytotoxicity of metal NPs is that oxidative stress is induced by an ROS burst. Exposure to NPs is followed by intracellular ROS production, which may sharply increase, thus inducing an ROS burst in cells [183]. For metal-based NPs demonstrating robust antibacterial activity, the conversion and recycling of valence states of multivalent metals are crucial in the production of ROS. The metal ions released by NPs participate in redox cycling and chemocatalysis via the Fenton reaction. Fenton-like oxidation is a set of radical reaction processes in which a redox reaction may occur with the participation of multivalent metal ions and hydrogen peroxide to yield radicals. In Fenton/Fenton-like oxidation systems exploiting the dual roles of H_2_O_2_ as a reductant and an oxidant, multivalent metal cations (including Fe and Cu) are important catalysts, which can activate H_2_O_2_ to generate ROS in the process mediated by both lower-valent and higher-valent metal oxidation states, owing to their reduction and oxidation properties, respectively [190]. The conventional Fenton reaction is initiated by interactions between hydrogen peroxide and Fe cations (Figure 6).

It should be noted that the equation for the silver system is not a simple one-step stoichiometric acid-catalyzed reaction. Gaps still exist in our mechanistic understanding of the silver(I) cation’s reduction process. Electron spin resonance (ESR) spectroscopy coupled with spin trapping and spin labeling is recognized as the most reliable and direct method for identifying and quantifying short-lived free radicals. ESR was used to provide direct evidence of ROS generation during decomposition of H_2_O_2_ assisted by AgNPs [191]. Hydroxyl radical formation was observed under acidic conditions and was accompanied by dissolution of AgNPs. In contrast, O_2_ evolution was observed in alkaline solutions containing H_2_O_2_ and AgNPs; however, no net dissolution of AgNPs was observed due to the re-reduction of Ag^+^, as evidenced by a cyclic reaction. This work examining the effects of AgNPs on ROS generation has pioneered a pivotal extension of mechanistic information on the effects of AgNPs on the oxidation/reduction of H_2_O_2_, especially over a wide pH range. In summary, it was demonstrated that AgNPs can induce •OH and O_2_ production in the presence of H_2_O_2_. The generation of hydroxyl radicals and oxygen was strongly dependent on pH, based on a Fenton-like reaction in an acidic environment and a redox reaction in an alkaline environment, respectively.

Excess copper is a universal toxicant [192]. A long-standing hypothesis for the mechanism by which copper poisons cells is that copper reacts with endogenous hydrogen peroxide to generate hydroxyl radicals in a process analogous to the Fenton reaction, triggering damage to lipids, proteins, and DNA and affecting cell viability [184]. In the face of emerging drug-resistant bacteria, copper-based nanomaterials hold great promise for combating microbial infections [137]. These metal oxide NPs possess dual character owing to their antioxidant and ROS-generating activity. As a potent antimicrobial, copper oxide efficiently kills bacteria and fungal pathogens due to its exhibited marked toxicity, especially at high concentrations [193]. At the same time, copper oxide may demonstrate an advantage in oxidative stress management through the effective inhibition of ROS [194]. The antibacterial mechanism of copper oxide NPs involves ROS production, supported by the tendency of Cu-containing NPs to alternate between cupric Cu(II) and cuprous Cu(I) oxidation states, making it unique from other metal NPs [195]. Consequently, it seems a clear possibility that copper generates hydroxyl radicals by participating in Fenton-like chemistry.

**Figure 7:**
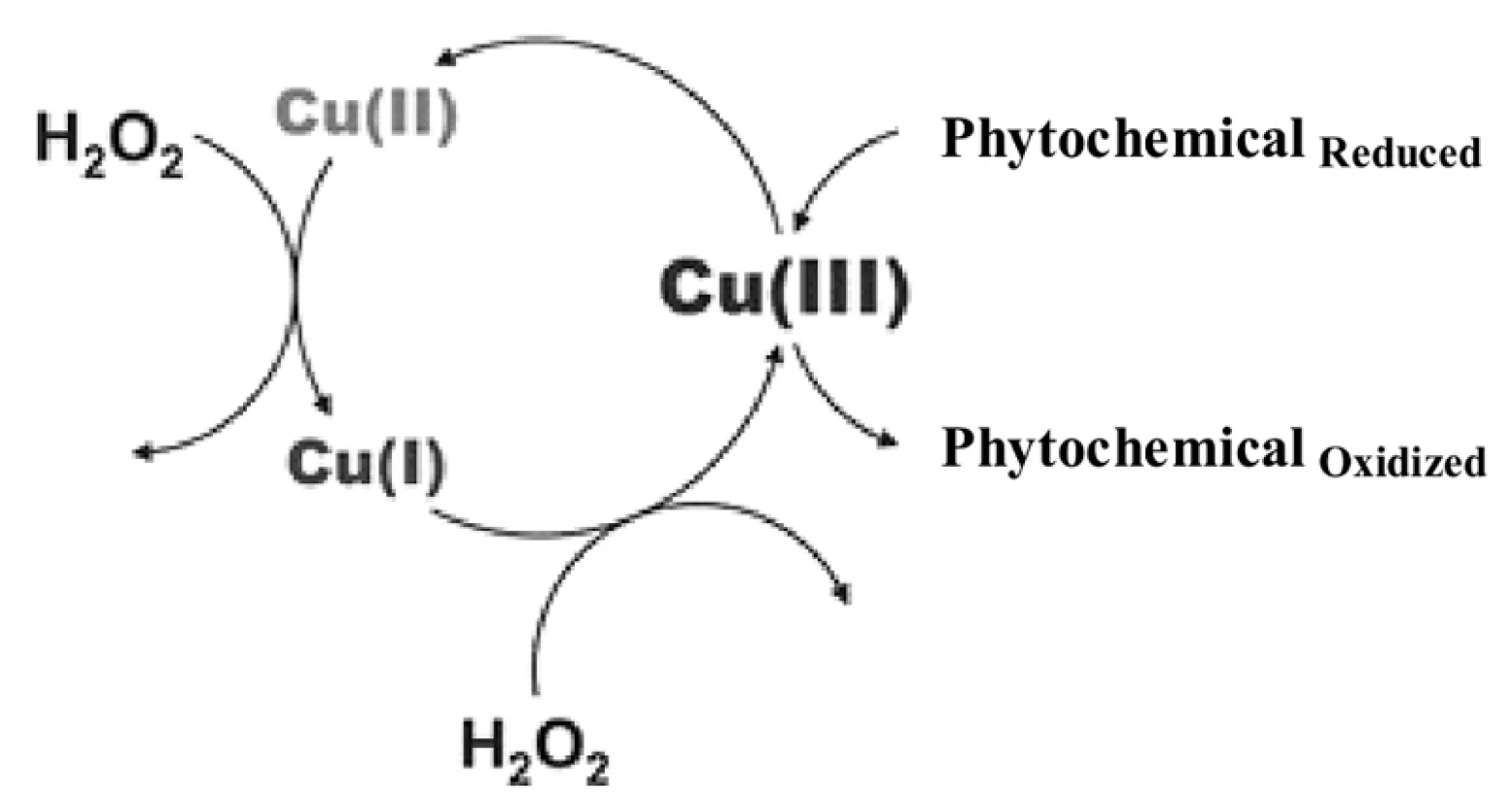
Dual oxidation strategy in Cu(II)/H_2_O_2_ system through the active Cu(III) intermediate.

The Fenton reaction for copper has been investigated widely and has experienced rapid development toward the recognition of Cu(III) as the primary and selective intermediate oxidant. The Cu(II)/H_2_O_2_ system is known for its unique dual oxidation strategy, resulting in a high potential to inactivate harmful microorganisms [196] (Figure 7).

Liu and Wang [190] noticed that under different reaction conditions, particularly at high H_2_O_2_ levels, the decay of Cu(I) was initially rapid. However, as the Cu(II) concentration increased with time, the back reduction of Cu(II) by H_2_O_2_ became more appreciable, the rate of Cu(I) decay was retarded, and the Cu(I) regenerated led to a lower apparent oxidation rate. The reaction between Cu(I) and H_2_O_2_ did not result in the production of •OH but involved the formation of a higher oxidation state of copper, Cu(III) [197]. The rate of •OH formation from the dissociation of Cu(III) was extremely low at pH 8.0, supporting the statement that •OH radicals were not an important oxidant in this system.

The rapid reaction rate of Cu(III) with Cu(I) contributes significantly to the redox cycle of copper. Cu-catalyzed production of Cu(III), a powerful oxidant, is discussed as being key to the oxidizing capacity of the Cu(I)/Cu(II)/H_2_O_2_/O_2_ system with exogenous hydrogen peroxide [196]. Hydrogen peroxide is able to oxidize and reduce copper, allowing it to catalytically degrade H_2_O_2_ (Figure 7). Cupryl species Cu(III) is formed during the Cu(I)–H_2_O_2_ reaction. Here, the primary oxidant responsible is the trivalent copper ion, not hydroxyl radical, as evidenced by the results of various analytical techniques reliably describing these interactions [198].

Defect-rich cuprous oxide (D-Cu_2_O) nanospheres were applied as a Fenton-like catalyst to generate Cu(III) [199] for the inactivation of antibiotic-resistant bacteria. D-Cu_2_O substantially enhanced Cu(III) formation when reacted with H_2_O_2_ compared to control Cu_2_O. The copper atoms in a lower valent state in D-Cu_2_O enhance the adsorption of hydrogen peroxide by improving the structural accessibility of adjacent oxygen atoms. This facilitates electron transfer processes and promotes subsequent Cu(III) generation [199].

Although several works emphasize the role of trivalent copper (Cu(III)/cupryl species) as the primary oxidant in Cu-catalyzed systems, recent advanced oxidation studies suggest that Cu(III) is involved in specific pH and ligand-binding conditions. Therefore, stating this as a universal rule for all copper-based antibacterial nanomaterials is incorrect. Metal-mediated radical generation involves complex surface redox dynamics or indirect release of iron via iron–sulfur cluster disruption. Under physiological and environmental conditions, hydroxyl radicals and superoxide radical anions remain the primary cytotoxic drivers. Excessive levels of superoxide and hydrogen peroxide can disrupt several amino acid biosynthetic pathways in bacteria. Copper inactivates iron–sulfur cluster dehydratases inside bacterial cells. ROS block branched-chain amino acid biosynthesis by directly damaging the iron–sulfur clusters of the dehydratases, first of all, dihydroxy acid dehydratase in the common branched-chain amino acid pathway, and then isopropylmalate isomerase (IPMI) in the leucine-specific branch [192]. The discovery that copper destroys iron–sulfur clusters testifies to another possibility: that the released iron atoms overload bacterial cells and proportionately accelerate Fe-based Fenton chemistry. Consistent with these ideas, workers have shown that iron chelators can suppress some aspects of copper toxicity.

### 5.6. Dual ROS and Non-ROS Mechanism of MgONPs Against Bacteria

It has been reported that the antibacterial activity of MgONPs is attributed to the production of ROS, which induce lipid peroxidation in bacteria [200]. In contrast, the authors of one study also found non-ROS-mediated bacterial toxicity in MgO nanoparticles, suggesting oxidative stress might not be the primary mechanism of cell death [149]. These authors found that despite the lack of ROS generation detected with reliable measurements, all of the studied MgO samples exhibited robust antibacterial activity. The authors suggested that the Mg^2+^ cations released from MgONPs played a decisive role in the antibacterial effect observed. However, neither a combination of Mg ions and increased pH nor the supernatant solution after MgONP removal resulted in as considerable a decrease in the bacterial colony amount as exposure to MgONPs, indicating that other mechanisms contribute to antibacterial activity.

**Figure 8:**
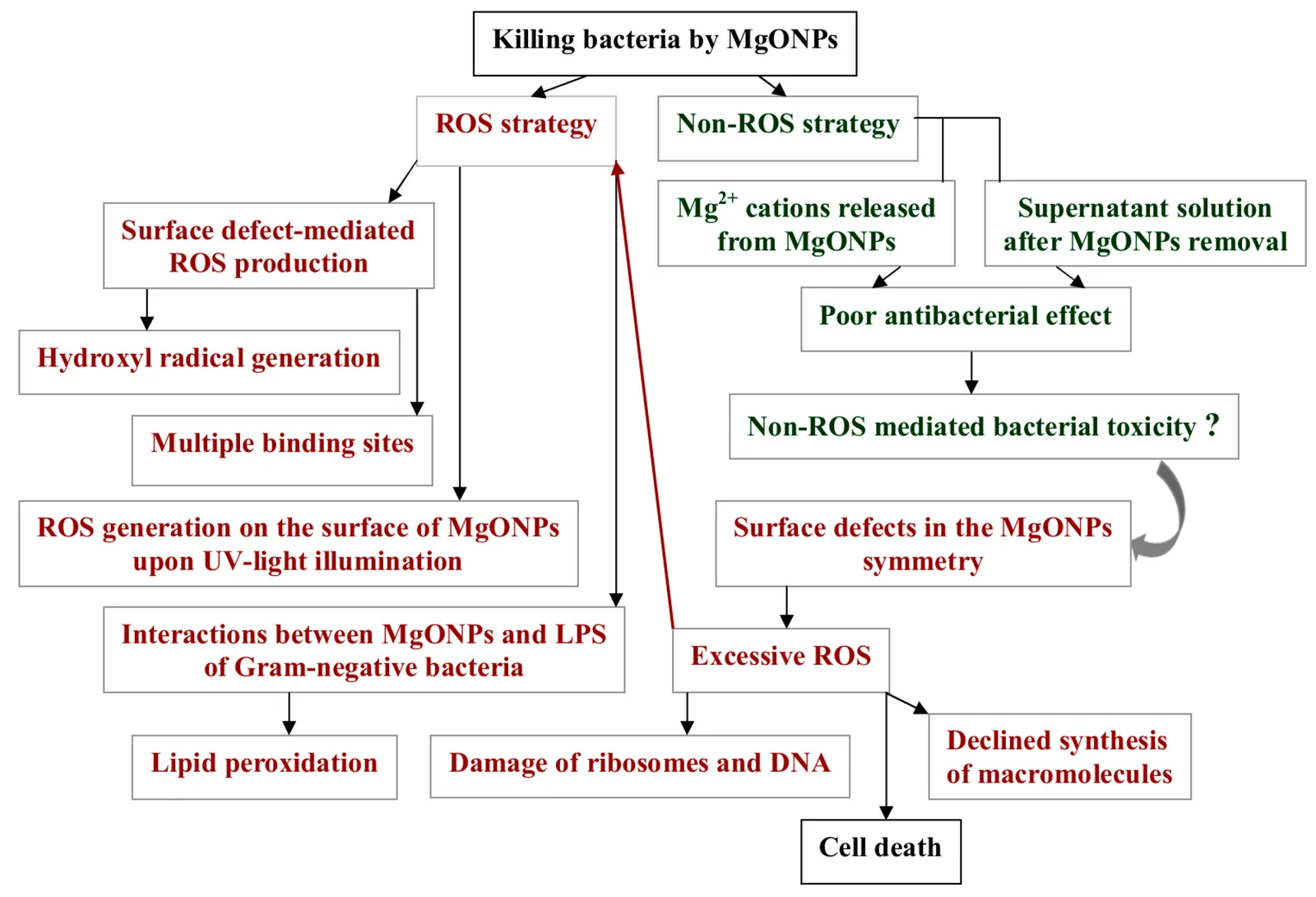
Multiple mechanisms underlying the action of MgONPs on bacteria.

The issue is that surface defects in MgONP symmetry can also account for antibacterial potential. Hydroxyl radical generation was observed in one series of MgO samples with the highest concentration of surface defects among the samples examined by Leung et al. [149]. Ultimately, the authors concluded on the possibility that the mechanism of cell damage is the attachment of MgO material to cell membrane chemical components followed by membrane mechanical injury. These interactions could be electrostatic. In addition, MgONPs likely contain multiple binding sites suitable for several types of chemical functional groups on the bound molecules. Appreciable interactions between MgONPs and LPSs on the outer membrane of Gram-negative bacteria are observed, contributing to the putative mechanism underlying the antibacterial action of these metal oxide NPs. Illumination, the presence of Mg cations, and a change in pH all have additional effects, which further enhance antibacterial activity [149] (Figure 8).

The limited antibacterial activity of MgO nanomaterials is one of the main bottlenecks to realizing their large-scale antibacterial application [201]. The antibacterial properties of nanostructured MgO are strongly dependent on surface defect-mediated ROS production. Electron paramagnetic resonance (EPR) spectroscopy, a powerful tool for the in-depth characterization of oxygen radicals and their interaction with solid surfaces, showed that charged oxygen species were generated on the surface of MgONPs upon UV light illumination [202]. The specific spectroscopic property changes in the MgO nanocrystals were indicative of charge separation effects at the surfaces and interfaces of the MgO nanograins. When excited with sufficient energy, MgO nanostructures can undergo charge separation and charge transfer to molecular oxygen and other adsorbates adsorbed at MgO nanocrystal surfaces. However, a full understanding of how charged oxygen particles are created and how they interact with the MgONP surface is still lacking.

There exists a complex interplay between superoxide anion radicals in surface-adsorbed form and different types of water molecules and hydroxyls that form upon surface hydration up to water film formation around metal oxide grains. Intergranular radical processes are currently poorly understood. Molecular oxygen likely acts as a scavenger of photogenerated surface electrons. However, oxygen radicals adsorbed at MgO nanocrystal surfaces are characterized by limited chemical stability in the presence of interfacial water and convert into UV−vis silent diamagnetic products under ambient conditions. EPR spectroscopy has been used to model this process, and the outcomes suggest the reactivity of intergranular oxygen radicals toward water vapor. The first steps may involve the protonation of superoxide anions followed by the transfer of a hydrogen atom originating either from another water molecule or from surface hydroxyls to the hydroperoxyl radical to yield hydrogen peroxide and a highly reactive hydroxyl radical. Other reaction steps may occur in parallel.

### 5.7. Toxicity Issues Inherent in Bioproduced Metal NPs

The adverse effects of NPs are not limited to pathogenic bacteria. Therefore, the regulatory framework and the potential risks associated with the use of NPs should be taken into consideration when discussing antibacterial nanocomposition manufacturing. Despite their green synthesis origin, biogenic NPs exhibit various toxicity mechanisms that require careful consideration. Metal NPs demonstrate toxicity in both bacterial cells and animal models, with specific molecular mechanisms varying between different NP types [203]. The primary toxicity pathway involves oxidative stress through the generation of ROS, which affects ecological receptors and organisms. Many works specifically address the nanotoxicology of biogenic metal oxide nanoparticles, noting that while these materials show increasing importance, comprehensive toxicity characterization remains necessary for nanosafety considerations [204].

The toxicity mechanisms include effects linked to the release of free metal ions, dissolution-enhanced toxicity, and direct intercalation with biological targets. The environmental behavior of biogenic nanocatalysts significantly influences their toxicity profiles. NPs undergo substantial physicochemical transformations in environmental conditions, with ionic strength, pH, and surface potential strongly affecting their stability and aggregation [204]. These transformations directly impact bioavailability and aging processes, including surface coating effects and interactions with dissolved organic carbon. There are also concerns regarding the accumulation, translocation, and biotransformation of nanocatalysts in food cycles, particularly affecting plants and fish [205]. This bioaccumulation potential represents a significant environmental risk that must be considered in the deployment of biogenic metal NPs for water treatment and environmental remediation applications.

Oyewole et al. [165] note that the cytotoxic effects of biogenic metallic nanoparticles result from their physicochemical properties, but these effects can be mitigated through the use of appropriate capping agents that serve as reducing and stabilizing agents. This finding could suggest potential strategies for designing safer plant-derived metal NPs. However, when implementing this nanomaterial to eradicate bacterial phytopathogens, overusing NPs can lead to high-dose accumulation in food chains, causing high risk. Furthermore, the generation of ROS must be addressed with caution, as these species are not necessarily selective for pathogenic bacteria and may affect non-target cells and organisms. Care should be taken to prevent the toxic side effects of NPs before marketing nano-agriproducts [206]. In general, the inherent complications of phytosynthesis, including safe condition selection, process parameter optimization, bulk production scaling, physicochemical control, nanomaterial cytotoxicity assessment, and knowledge deficiencies, currently trap green synthesis largely in the laboratory phase [207].

## 6. Conclusive Remarks

The emergent antibiotic resistance of pathogenic bacteria necessitates rapid development to overtake new drug discovery. To address this microbial resistance, engineered metal-containing NPs show significant potential. Plant extracts are a convenient and eco-friendly way to improve the nanobiotechnological biofabrication of metal NPs. Botanical bactericidal substances conjugated with nanostructures could assist in identifying a way to develop alternative antibacterials. The key mechanisms behind the plant-mediated synthesis of NPs include phyto-assisted reduction and stabilization processes that yield metal-containing nanostructures harnessing ROS to combat bacterial pathogens. It is imperative to understand how phytochemically produced nanomaterials, including NPs, exert regulatory effects on bacteria and how cellular toxicity caused by ROS production affects bacterial pathogens. Depending on the plant source and inorganic precursor used, each method for obtaining metal NPs has both benefits and drawbacks of its own. Different facets of the role that metal NPs play in antibacterial applications, including compromised selectivity of ROS for bacterial pathogens, should be considered as forming an inseparable whole with their environmental toxicity.

## Figures and Tables

**Table 1:** Selected metal-containing NPs functioning via ROS induction-related antimicrobial properties.

Nanoparticle Type	Nanoparticle Size, nm	Level or MIC, µg/mL	Plant	Bacterial Pathogen	Reference
Ag	143.5	90	*Allium cepa*	*Pseudomonas syringe*	[7]
Ag	143.5	70	*Allium cepa*	*Erwinia* sp.	[7]
Ag	143.5	130	*Allium cepa*	*Bacillus megaterium*	[7]
Ag	ND *	90	*Allium sativum*	*Pseudomonas syringe*	[7]
Ag	ND	90	*Allium sativum*	*Erwinia* sp.	[7]
Ag	ND	110	*Allium sativum*	*Bacillus megaterium*	[7]
Ag	18–33	1.5	*Hyphaene thebaica*	*Staphylococcus aureus* ATCC 29213	[17]
Ag	19.8–92.8	20	*Phyllanthus emblica*	*Acidovorax oryzae* RS-2	[18]
Ag	ND	ND	*Phyllanthus pinnatus*	*Bacillus subtilis*, *Mycobacterium smegmatis*, *Proteus vulgaris*, *Pseudomonas aeruginosa*, *Shigella flexneri*, *Vibrio cholera*	[19]
Ag	15–80	100–400	*Phyllanthus urinaria*	*Escherichia coli*, *Proteus mirabilis, Pseudomonas aeruginosa*, *Salmonella typhi*, *Vibrio cholera*,	[20]
Ag	5–55	100	*Pongamia pinnata*	*Klebsiella planticola*	[8]
Ag	5–55	100	*Pongamia pinnata*	*Staphylococcus aureus*	[8]
Ag	15	25	*Sterculia diversifolia*	*Escherichia coli* ATCC 8739, *Staphylococcus aureus* ATCC 6538	[21]
Ag	19.401	83	*Urtica diocia*	*Enterococcus faecalis*	[22]
Ag	19.401	36–75	*Urtica diocia*	*Escherichia coli*	[22]
Ag	19.401	38	*Urtica diocia*	*Staphylococcus epidermidis*	[22]
Ag	ND	110	*Zingiber officinale*	*Pseudomonas syringe*	[7]
Ag	ND	70	*Zingiber officinale*	*Erwinia* sp.	[7]
Ag	ND	110	*Zingiber officinale*	*Bacillus megaterium*	[7]
Ag_2_O	ND	ND	*Cyathea nilgiriensis*	*S. aureus*, *B. subtilis*, *E. coli*, *K. pneumoniae*, *M. luteus*, *S. paratyphi*	[23]
Cu	80	50	*Ageratum houstonianum*	*Escherichia coli* (MTCC no. 40)	[24]
Cu	63.46	50–250	*Cocculus hirsutus*	*Bacillus subtilis*, *Staphylococcus aureus*	[25]
CuO	ND	25–100	*Abutilon indicum*	*Escherichia coli*, *Salmonella typhi*, *Bacillus subtilis*, *Staphylococcus aureus*	[26]
CuO	ND	25–150	*Cedrus deodara*	*Escherichia coli*, *Staphylococcus aureus*	[27]
CuO	ND	ND	*Gloriosa superba*	*K. aerogenes*, *E. coli*, *S. aureus*, *P. desmolyticum*	[28]
CuO	Nanorods 90 × (400–500)	50–100	*Momordica charantia*	*Staphylococcus aureus*, *Streptococcus viridians*, *Staphylococcus epidermidis*, *Corynebacterium xerosis*, *Bacillus cereus*, *Escherichia coli*, *Pseudomonas aeruginosa*, *Proteus vulgaris*	[29]
CuO	20	ND	*Phyllanthus amarus*	*E. coli*, *P. aeruginosa*, *B. subtilis*, *S. aureus*	[30]
CuO	29.5–60.5	ND	*Saccharum officinarum*	*E. coli*, *P. aeruginosa*, *S. aureus*, *B. subtilis*	[31]
MgO	10.9	3.5	ND	*Xanthomonas oryzae* pv. *oryzae*	[32]
MnO_2_	18.8	3.6	ND	*Xanthomonas oryzae* pv. *oryzae*	[32]
TiO_2_	4–15	ND	*Phyllanthus niruri*, *Plectranthus amboinicus*	*Streptococcus mutans*	[33]
TiO_2_, TiO_2_/Zn	7	500–800	ND	*Xanthomonas* sp.	[15]
TiO_2_, ZnO	ND	50	*Citrus limon*	*Dickeya dadantii*	[34]
ZnO	62.8	3.5	ND	*Xanthomonas oryzae* pv. *oryzae*	[32]
ZnO	100	1600	ND	*Bacillus thuringiensis*, *Bacillus megaterium*	[35]
ZnO	25.96	ND	*Phyllanthus embilica*	*Klebsiella phnemonea*, *Salmonella typhi*	[36]
ZnO	52.50–57.75	1250	*Punica granatum*	*Staphylococcus aureus*, *Enterococcus faecalis*, *Escherichia coli*, *Enterococcus faecium*, *Listeria monocytogenes*, *Klebsiella pneumoniae*, *Streptococcus pneumoniae*, *Salmonella typhi*	[37]
ZnO	52.50–57.75	about 1.0	*Punica granatum*	*Bacillus cereus*, *Moraxella catarrhalis*, *Pseudomonas aeruginosa*, *Aeromonas hydrophila*	[37]

* ND means Not Determined.

**Table 2:** Plant metabolites as sources of antibacterials.

Plant Source of Antibacterials	Bacterial Pathogens	MIC µg/mL	Activity Attributed to Phytochemicals	Reference
*Aloe ferox*	*Streptococcus pyogenes*	200	Alkaloids, anthraquinones, pre-anthraquinones, anthrones, bianthraquinoids, chromones, flavonoids, coumarins, pyrenes	[60]
*Artemisia vulgaris*	*Streptococcus pyogenes*	391	ND *	[61]
*Cannabis sativa*	*Enterococcus faecalis*	13.01	Myricetin, naringenin, apigenin, luteolin, rutin	[62]
*Cinnamomum camphora*	ND	ND	Monoterpene 1,8-cineole (1,3,3-trimethyl-2-oxabicyclo [2.2.2]octane), known as eucalyptol	[63]
*Curcuma longa*	*Bacillus subtilis*	6.50	ND	[64]
*Curcuma longa*	*Staphylococcus aureus*	6.77	ND	[64]
*Curcuma longa*	*Klebsiella pneumoniae*	6.8	ND	[64]
*Curcuma longa*	*Pseudomonas aeruginosa*	7.27	ND	[64]
*Curcuma longa*	*Escherichia coli*	7.58	ND	[64]
*Glycyrhhiza glabra*	*Pseudomonas aeruginosa*	10	Flavonoids, tannins, carbohydrates, glycosides, steroids, terpenoids, alkaloids, saponins	[65]
*Eucalyptus globulus*	ND	ND	Monoterpene 1,8-cineole (1,3,3-trimethyl-2-oxabicyclo [2.2.2]octane), known as eucalyptol	[63]
*Eucalyptus nicholii*	ND	ND	Monoterpene 1,8-cineole (1,3,3-trimethyl-2-oxabicyclo [2.2.2]octane), known as eucalyptol	[66]
*Lavandula stoechas*	*Salmonella typhimurium*	35.9	Monoterpene (-)-fenchone (1,3,3-trimethyl bicyclo [2.2.1]heptane-2-one), camphor(+)−2-bornanone	[67]
*Lavandula stoechas*	*Enterococcus faecalis*	35.9	Monoterpene (-)-fenchone (1,3,3-trimethyl bicyclo [2.2.1]heptane-2-one), camphor(+)−2-bornanone	[67]
*Lavandula stoechas*	*Listeria monocytogenes*	35.9	Monoterpene (-)-fenchone (1,3,3-trimethyl bicyclo [2.2.1]heptane-2-one), camphor(+)−2-bornanone	[67]
*Lavandula pedunculata*	*Listeria monocytogenes*	100	Rosmarinic acid, salvianolic acid B, caffeic acid, luteolin-7-oglucuronide	[68]
*Mentha piperita*	ND	ND	(−)-Menthol, with smaller amounts of (+)-*neo*-menthol and (+)-*iso*-menthol	[69]
*Mentha viridis*	ND	ND	Monoterpene menthol	[70]
*Ocimum gratissimum*	*Staphylococcus aureus*	5	β-Selinene, γ-terpinene, caryophyllene oxide, trans-caryophyllene, sabinene, α-copaene, α-humulene, trans-phytol, 2-isopropyl-5-methylphenol (thymol)	[71]
*Ocimum gratissimum*	*Bacillus cereus*	10	Cymene, glycerol, β-selinene, 3-(1,1-dimethylethyl)−4-methoxyphenol, 2-methyl-5-(1-methylethyl)-phenol (carvacrol), trans-phytol, 2-isopropyl-5-methylphenol (thymol)	[71]
*Ocimum gratissimum*	*Escherichia coli*	20	Cymene, glycerol, β-selinene, 3-(1,1-dimethylethyl)−4-methoxyphenol, 2-methyl-5-(1-methylethyl)-phenol (carvacrol), trans-phytol, 2-isopropyl-5-methylphenol (thymol)	[71]
*Ocimum gratissimum*	*Salmonella typhimurium*	40	Cymene, glycerol, β-selinene, 3-(1,1-dimethylethyl)−4-methoxyphenol, 2-methyl-5-(1-methylethyl)-phenol (carvacrol), trans-phytol, 2-isopropyl-5-methylphenol (thymol)	[71]
*Piper umbellatum*	*Salmonella typhimurium*	12.5	Flavonoids, alkaloids, steroids, terpenes	[72]
*Piper umbellatum*	*Enterococcus faecalis*	12.5	Flavonoids, alkaloids, steroids, terpenes	[72]
*Piper umbellatum*	*Streptococcus pyogenes*	200	Flavonoids, alkaloids, steroids, terpenes	[72]
*Quercus coccifera*	*Bacillus cereus*	0.5	Tannins, polydatin, kermesoside, cocciferoside, (-)-8-chlorocatechin	[73]
*Quercus coccifera*	*Bacillus subtilis*	1	Tannins, polydatin, kermesoside, cocciferoside, (-)-8-chlorocatechin	[73]
*Quercus coccifera*	*Pseudomonas aeruginosa*	4	Tannins, polydatin, kermesoside, cocciferoside, (-)-8-chlorocatechin	[73]
*Quercus coccifera*	*Klebsiella pneumoniae*	16	Tannins, polydatin, kermesoside, cocciferoside, (-)−8-chlorocatechin	[73]
*Rosmarinus officinalis*	ND	ND	Monoterpene 1,8-cineole (1,3,3-trimethyl-2-oxabicyclo [2.2.2]octane), known as eucalyptol	[63]
*Santalum album*	ND	ND	Oxygenated sesquiterpenes (Z)-α-santalol, (Z)-β-santalol	[74]
*Solanum xanthocarpum*	*Staphylococcus aureus*	ND	Alpha-linoleic acid, palmitic acid	[75]
*Tamarix gallica*	*Listeria monocytogenes*	146	Quercetin, quercetin 3-O-glucuronide, flavone, (−)-epicatechin, kaempferol 3-O-β-D-glucuronide, resveratrol 3-O-glucoside	[76]
*Tinospora cordifolia*	*Staphylococcus aureus*	7.50	ND	[64]
*Zingiber officinale*	*Bacillus subtilis*	8.17	ND	[64]
*Zingiber officinale*	*Pseudomonas aeruginosa*	10	Flavonoids, tannins, steroids, terpenoids, alkaloids, saponins	[65]
*Zingiber officinale*	*Escherichia coli*	13.33	ND	[64]
*Zingiber officinale*	*Klebsiella pneumoniae*	15.8	ND	[64]

* ND means Not Determined.

## Data Availability

No new data were created or analyzed in this study. Data sharing is not applicable to this article.

## Abbreviations

The following abbreviations are used in this manuscript:

GSH	Reduced glutathione
MIC	Minimum inhibitory concentration
MNPs	Metal nanoparticles
NPs	Nanoparticles
ROS	Reactive oxygen species
